# Impact of the absence of opioid anesthesia on postoperative outcome indicators: a systematic review and meta-analysis

**DOI:** 10.3389/fmed.2025.1639968

**Published:** 2025-08-18

**Authors:** Jiarun Qin, Jialei Zhang, Jianing Bo, Xiaoyan Ma, Xiaofeng He

**Affiliations:** ^1^Department of Anesthesiology, Changzhi People‘s Hospital Affiliated to Changzhi Medical College, Changzhi, China; ^2^Department of Pain Management, Changzhi People‘s Hospital Affiliated to Changzhi Medical College, Changzhi, China; ^3^Institute of Evidence-Based Medicine, Heping Hospital Affiliated to Changzhi Medical College, Changzhi, China

**Keywords:** opioid-free anesthesia, opioid drugs, postoperative recovery, systematic review, meta-analysis

## Abstract

**Objective:**

This study aimed to examine the effect of opioid-free anesthesia (OFA) on postoperative outcome indicators and explore its application in thoracoscopic or laparoscopic as well as non-thoracoscopic or laparoscopic surgeries, providing a scientific basis for clinical decision-making.

**Method:**

A systematic search was conducted for clinical studies comparing OFA and opioid-based anesthesia (OBA) published from the establishment of the databases to May 2025 using databases such as PubMed, Web of Science, Embase, and Cochrane Library. The primary outcome was the incidence of postoperative nausea and vomiting (PONV). Secondary outcomes included perioperative recovery indicators, the need for postoperative emergency analgesia, postoperative pain score (VAS, NRS), and adverse reactions.

**Results:**

A total of 3,766 relevant studies were initially identified, and 68 randomized controlled trials involving 5,426 patients were ultimately included. Compared with OBA, OFA significantly reduced the risks of PONV (RR = 0.50, 95% CI: 0.39–0.64), nausea alone (RR = 0.34, 95% CI: 0.25–0.46), vomiting alone (RR = 0.34, 95% CI: 0.25–0.46), and the need for postoperative emergency analgesia (RR = 0.61, 95% CI: 0.51–0.72). OFA was also associated with lower 24 h postoperative NRS pain scores (SMD = −0.32, 95% CI: −0.53 to −0.10). For outcomes with high heterogeneity (*I*^2^ > 75%), the systematic review showed that most studies did not find a significant reduction in postoperative VAS pain scores with OFA. However, over two-thirds of the studies have shown that OFA can improve the quality of postoperative recovery (QoR-40). Approximately half of the studies suggested that OFA may prolong extubation time, while most found no significant difference in PACU stay time.

**Conclusion:**

In summary, OFA not only significantly reduces postoperative PONV, but also lowers the demand for analgesic drugs and improves the quality of postoperative recovery. However, its effect on some postoperative recovery indicators is limited, and further high-quality studies are required to confirm these findings. OFA is expected to serve as a safe and effective anesthesia strategy to optimize the perioperative outcomes of patients.

## 1 Introduction

The enhanced recovery after surgery (ERAS) program has gained significant attention (1) and is widely used in perioperative management across various surgical specialties. Opioids, the cornerstone of perioperative analgesia, are associated with adverse effects such as intestinal motility inhibition, sedation, respiratory depression, urinary retention, postoperative nausea and vomiting (PONV), pruritus, and hyperalgesia (2). Additionally, opioids can exacerbate sleep apnea and increase the risk of critical respiratory events (CREs) during postoperative recovery, posing a serious threat to patient safety (3). Opioid-free anesthesia (OFA) is a multimodal approach that aims to eliminate the use of opioids throughout the perioperative period by integrating various strategies, thereby reducing opioid-related adverse effects while maintaining patient comfort (4). The increasing adoption of OFA reflects a growing response to the risks associated with opioid adverse drug events. However, its application remains highly controversial. Given the inconsistent findings across studies, further investigation is warranted. Currently, there is a lack of unified guidelines for implementing OFA. Clinicians' attitudes toward OFA are characterized by a coexistence of active exploration and cautious implementation, largely due to the absence of high-level evidence. Consequently, the benefits and drawbacks of OFA remain a matter of uncertainty for clinicians. Several systematic reviews and meta-analyses have examined the impact of OFA on postoperative recovery, analgesic efficacy, and opioid-related side effects. For example, Minke (5) conducted a systematic review summarizing the effects of OFA on both acute and chronic postoperative pain. However, significant heterogeneity exists in the research on OFA, and the level of evidence is generally medium to low (6). The external validity of OFA in improving postoperative recovery remains to be further confirmed. These studies offer preliminary evidence supporting the clinical application of OFA. Nevertheless, given the continuous emergence of new research, some recent high-quality randomized controlled trials (RCTs) have not been included in previous analyses, and earlier meta-analyses are often limited by small sample sizes and high heterogeneity. While OFA has attracted attention for its potential to reduce opioid-related adverse effects, the conclusions of prior studies have been inconsistent. Therefore, we conducted this meta-analysis. Based on the latest literature, we systematically reviewed all available evidence regarding opioid and OFA and their impacts on postoperative outcomes, aiming to provide more comprehensive, up-to-date, and high-quality evidence for clinical practice.

In this study, we strictly adhered to the PICOS framework to ensure transparency and reproducibility. Specifically, the population (P) included surgical patients undergoing general anesthesia with ASA physical status I to III. The intervention (I) was OFA, defined as complete avoidance of opioid use during the perioperative period. The comparator (C) was conventional opioid-based anesthesia (OBA). The primary outcome (O) was the incidence of PONV, while secondary outcomes included quality of recovery (QoR-40 score), postoperative emergency analgesic requirement, adverse events, length of hospital stay, postoperative pain scores (NRS or VAS), extubation time, and post-anesthesia care unit (PACU) stay duration. The study design (S) consisted of a systematic review and meta-analysis, including RCTs published up to May 2025, following the PRISMA guidelines.

## 2 Methods

This study was conducted in accordance with the checklist recommended by the Preferred Reporting Items for Systematic Reviews and Meta-Analysis (PRISMA) (7).

### 2.1 Research objects and retrieval strategies

The research subjects were patients receiving general anesthesia. Databases such as PubMed, Web of Science, Embase, and Cochrane Library were systematically retrieved to comprehensively collect studies that met the inclusion criteria. The retrieval time ranged from the establishment of the databases to May 2025 to ensure the coverage of the latest research results. The search terms are as follows: non-opioid, opioid-free, general anesthesia, randomized controlled trial.

### 2.2 Inclusion and exclusion criteria

Inclusion criteria: (1) Surgical patients receiving general anesthesia, with ASA grades I–III; (2) Compare anesthesia without opioid anesthetic drugs with that containing opioid anesthetic drugs; (3) The research design stipulates the sample size; Exclusion criteria: (1) The research lacks original data or has incomplete materials, making it impossible to extract the data; (2) Secondary studies such as meta-analysis, case reports, reviews, abstracts, and non-original literature studies; (3) Animal experiments, etc. This study ensured the homogeneity of the research subjects and the repeatability of the results through strict inclusion and exclusion criteria, thereby more reliably evaluating the impact of OFA on postoperative outcomes.

### 2.3 Data extraction

During the data extraction stage, a data extraction table is first created. Then, two researchers screened all the studies according to the inclusion and exclusion criteria and then extracted the relevant data from the studies for statistical analysis. If the two researchers arrive at different results and fail to reach a consensus after discussion, the third researcher will re-extract the data for analysis and obtain the results. Finally, the three researchers cross-reviewed and confirmed the accuracy of all the data. If the data in an article is ambiguous or controversial, contact the original author to obtain the accurate original data. The extracted research characteristics include: first author, publication year, country, number of patients, type of OBA protocol, type of anesthesia protocol in the OFA group, outcome indicators, and study type. The primary outcome measure was the incidence of PONV. If the study divided it into two indicators, nausea and vomiting, they would be studied separately. Secondary outcome measures included postoperative recovery quality (QoR-40 score), the incidence of requiring emergency analgesia, postoperative adverse reactions, perioperative recovery indicators, and pain score. If the pain score and QoR-40 score were reported at multiple time points after the operation, the scores at the time point closest to 24 h after the operation were collected for meta-analysis.

### 2.4 Quality assessment

To assess the risk bias in the included studies, this study used the risk bias assessment tool RoB 2 (8) (Risk of Bias 2) recommended by Cochrane to conduct a systematic review of all RCTS. Each assessment result is classified as “low risk,” “some concern,” or “high risk.” The assessment work was independently completed by two researchers. If there were any differences, the final judgment would be determined after the participation of a third researcher in the discussion.

### 2.5 Statistical analysis

A meta-analysis was performed using Stata 18.0 statistical software. Binary categorical variables were expressed as relative risk (RR) and 95% confidence interval (CI); If the included studies report the mean and standard deviation, the effect size is expressed as the mean difference. For studies involving three study groups, the means of the two study groups between the two OFA or OBA groups were estimated. For studies that use other descriptive methods, such as median and quartile ranges, the standardized mean difference is calculated. All the included studies were divided into two groups according to the surgical methods: the “thoracoscopic or laparoscopic group” and the “non-thoracoscopic or laparoscopic group.” The heterogeneity was evaluated by the Cochran *Q* test and the *I*^2^ value (9). A random-effects model was used for all analyses for two main reasons: (1) the Q test is characterized by low statistical power for between-study heterogeneity, which is especially relevant when few studies are available; (2) the random-effects model is a more conservative choice when heterogeneity is present, whereas it reduces to the fixed-effects model when heterogeneity is absent. *P* < 0.05 was considered statistically significant. When substantial heterogeneity is detected (*I*^2^ > 75%), a meta-analysis is deemed inappropriate. In such cases, a systematic review with qualitative synthesis is conducted instead (10). For all the analyses, *P* < 0.05 was considered statistically significant. Subgroup analyses were performed to evaluate the effects of OFA across different surgical types, including thoracoscopic surgery, laparoscopic surgery, and other non-laparoscopic/thoracoscopic procedures. Additional subgroup analyses were conducted based on anesthetic techniques, specifically comparing total intravenous anesthesia (TIVA) and combined intravenous-inhalation anesthesia. Furthermore, the impact of regional nerve block (RNB) use was also examined to assess its influence on postoperative outcomes within the OFA group. Then, the sensitivity analysis was conducted using the following two methods: (1) The elimination method was applied step-by-step, with each study, to observe whether the effect size changed; (2) Low-quality literature (“high-risk” or “some concerns”) is excluded, and a meta-analysis is re-conducted to explore the impact of low-quality studies on the overall effect. Publication bias was analyzed by using Begg's funnel plot (11) and Egger's test (12) when the number of studies was >10. If publication bias is detected, the “trim and fill method” should be used to adjust the results (13). Meta-regression analysis was performed to further explore the impact of clinical factors on postoperative outcomes. Variables included surgical type, anesthetic technique, and the use of regional nerve blocks. The analysis used a random-effects model to assess their impact on key outcomes, with results presented as regression coefficients and 95% confidence intervals.

## 3 Results

### 3.1 Literature search

**Figure 1:**
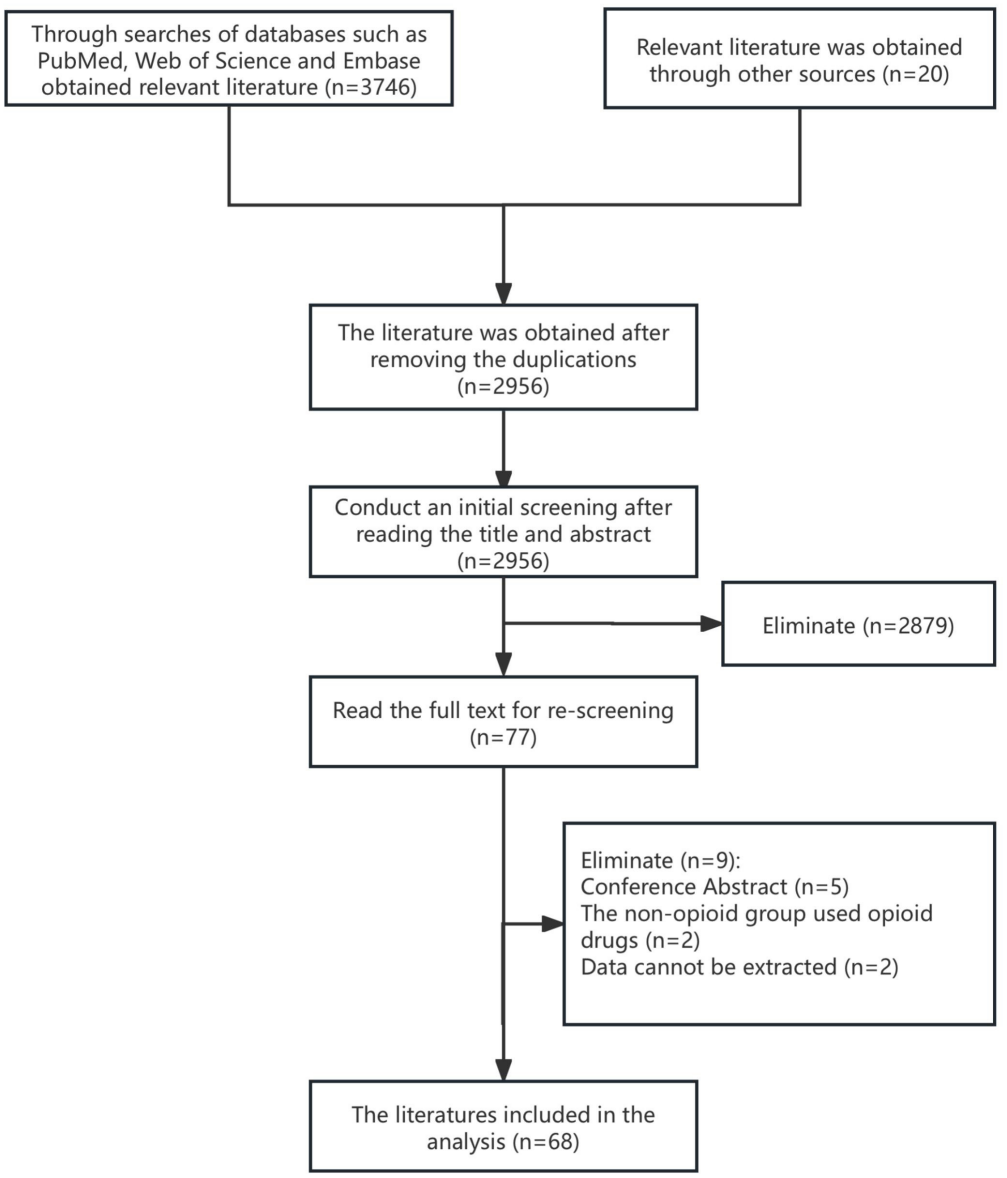
Flowchart of literature screening.

Through systematic retrieval, a total of 3,766 relevant studies were identified. After removing duplicates, the remaining articles were screened based on the predefined inclusion and exclusion criteria by thoroughly reviewing their titles, abstracts, and full-text contents. Ultimately, 68 studies were included in this analysis. For detailed information on the literature retrieval and study selection process, please refer to the Supplementary Table 1. A total of 5,426 patients participated in these studies, with 2,732 assigned to the experimental group and 2,694 to the control group. The characteristics of the included studies are summarized in Table 1, and the literature screening process is illustrated in Figure 1. The specific dosing regimen is detailed in Supplementary Table 2.

**Table 1:** Basic characteristics of the study.

**First author/year**	**Country**	**Population (Surgical type)**	**Sample size (OFA/OA)**	**Outcomes**	**Intraoperative regimen in the opioid–free anesthesia group**	**Intraoperative regimen in the control group**
Barakat (27) 2025	Lebanon	Laparoscopic sleeve gastrectomy	40/43	Opioid consumption during the post-anesthesia care unit (PACU); Intraoperative hemodynamic stability; Time to extubation; PACU stay duration; Opioid consumption during the first 48 h; Anti-emetic requirements	Dexmedetomidine, lidocaine, propofol, rocuronium, ketamine, sevofurane	Propofol, fentanyl, ketamine, rocuronium, remifentanil, sevofurane
Zeng (54) 2025	China	Tonsillectomy	22/22	Pain score; The time to first food ingestion, sleep quality, nausea, vomiting; Respiratory depression, insufficient analgesia, number of children requiring hydromorphone rescue analgesia, and differences in psychological symptoms; Postoperative bleeding, and caregiver satisfaction	Sevoflurane, propofol, cisatracurium, esketamine, dexmedetomidine	Sevoflurane, propofol, cisatracurium, fentanyl, remifentanil
Bao (1) 2024	China	Video-assisted thoracoscopic surgery	86/88	Incidence of PONV; PONV severity; Postoperative pain; Haemodynamic changes during anesthesia; Length of stay (LOS) in the recovery ward and hospital	Dexmedetomidine, dexamethasone, midazolam, propofol, rocuronium, lidocaine, magnesium sulfate, cisatracurium	Dexmedetomidine, dexamethasone, midazolam, propofol, rocuronium, sufentanil, propofol, remifentanil, cisatracurium
Chassery (14) 2024	France	Hip arthroplasty	40/40	The opioid consumption: median cumulative OME consumption in the PACU; Pain scores; Walking recovery time; Adverse events	Dexmedetomidine	Sufentanil
Copik (55) 2024	Poland	Video-assisted thoracic surgery	25/25	NRS and PHHPS; Total dose of postoperative oxycodone; Opioid related adverse events	Lidocaine, ketamine	Fentanyl
Leger (56) 2024	France	Major surgery	65/68	Early postoperative quality of recovery (Quality of Recovery-15); Quality of Recovery-15 at 48 and 72 h; Incidence of chronic pain; Quality of life at 3 months	Clonidine, magnesium sulfate, lidocaine, ketamine	Sufentanil, remifentanil, ketamine
Minqiang (57) 2024	China	Thoracoscopic sympathectomy	78/73	Perioperative complications; Vital signs, blood gas indices, visual analog scale (VAS) scores, adverse events, patient satisfaction	Propofol, dezocine, dexmedetomidine, intercostal nerve block	Propofol, fentanyl, cisatracurium, remifentanil
Ma (58) 2024	America	Arthroscopic temporomandibular joint surgery	30/30	The highest documented pain score; Perioperative opioid consumption, utilization, dosage, and timing of rescue analgesia; Postoperative nausea and vomiting in the PACU and at home; Pain satisfaction levels, occurrence of opioid-related adverse effects, duration of PACU and hospital stays; Total consumption of oxycodone-acetaminophen tablets	Lidocaine, ketamine, dexmedetomidine, sevoflurane	Fentanyl, sevoflurane
Bhardwaj (59) 2024	India	Breast cancer surgery	50/50	Comparison of analgesic efficacy; NRS pain scores; NLR and number of NKCs, T helper cells, cytotoxic T cells; Side effects	Propofol, cisatracurium, dexamethasone, sevofurane, dexmedetomidine, magnesium sulfate	Propofol, cisatracurium, dexamethasone, sevofurane, fentanyl
Wang (60) 2024	China	Thyroid and parathyroid surgery	197/197	Postoperative nausea and vomiting; Severity of PONV; Need for rescue anti-emetics; Need for rescue analgesics; Interventions for haemodynamic events; Desaturation after tracheal extubation; dizziness, headache, nightmare or hallucination; Time to tracheal extubation; duration of PACU and postoperative hospital stay; Patient satisfaction, rated using a 5-point Likert scale (ACS NSQIP)	Propofol, esketamine, lidocaine, dexmedetomidine, cisatracurium,	Propofol, sufentanil, lidocaine, cisatracurium
Zhou (61) 2024	China	Laparoscopic sleeve gastrectomy	35/36	Aantiemetic rescue; Pain scores, analgesic needs, extubation time, complications, the hemodynamic changes, and duration of hospital stay	Esketamine, dexmedetomidine, midazolam, propofol, rocuronium, sevoflurane, cisatracurium, TAP	Sufentanil, midazolam, propofol, rocuronium, sevoflurane, cisatracurium
Jose (36) 2023	India	Modified radical mastectomy	60/60	Intraoperative hemodynamic variables: Anesthetic requirement, Extubation response, Recovery profile	Propofol, lignocaine, succinylcholine, nitrous oxide in an oxygen mixture (66:33), atracurium, dexmedetomidine	Propofol, lignocaine, succinylcholine, nitrous oxide in an oxygen mixture (66:33), atracurium, morphine, bupivacaine
Cha (15) 2023	China	Hysteroscopy	45/45	The quality of recovery 24 h postoperatively (QoR-40 questionnaire); PONV; Time to extubation	Lidocaine, propofol, scoline	Sufentanil, propofol, scoline
Chen (62) 2023	China	Gynecological laparoscopic surgery	39/38	Visual Analog Scale (VAS); Intraoperative hemodynamic variables; Awakening and orientation recovery times; Number of postoperative rescue analgesia required; PONV; Pittsburgh Sleep Quality Index (PSQI) perioperatively	Esketamine, dexmedetomidine, TAP	Sufentanil, midazolam, propofol, cis-atracurium, TAP
Dai (63) 2023	China	Lower abdominal or pelvic surgery	62/20	BP, pulse oxygen saturation, reaction entropy, state entropy, and SPI values; Steward score; Dosage of propofol, dexmedetomidine, rocuronium, and diltiazem; Extubation time; and awake time	Propofol, rocuronium, QLB	Propofol, remifentanil, rocuronium, QLB
Elahwal (64) 2023	Egypt	Scoliosis surgery	25/25	Total postoperative morphine consumption at 24 h; Number of patients needed intraoperative magnesium; Time of first postoperative analgesic requirement; Visual analog scale (VAS); Side effects (PONV, hypotension, bradycardia, respiratory depression)	Midazolam, propofol, atracurium, dexmedetomidine, lidocaine, ketamine	Midazolam, propofol, atracurium, fentanyl
Krishnasamy Yuvaraj (35) 2023	India	Breast cancer surgeries	30/30	The quality of recovery in the postoperative period (QoR-40 score)	Dexmedetomidine, ketamine, lidocaine, propofol, vecuronium, D-SAPB	Fentanyl, propofol, vecuronium, D-SAPB
Liu (65) 2023	China	Thyroid surgery	33/33	Incidence of nausea, Incidence of vomiting, and the visual analog score (VAS) scores; The quality of recovery 40 40-questionnaire (QoR-40) scores	Dexmedetomidine, etomidate, ketamine, lidocaine, propofol, rocuronium	Etomidate, remifentanil, propofol, rocuronium
Orhon Ergun (66) 2023	Turkey	Video-assisted thoracoscopic surgery	37/37	Postoperative morphine requirement, postoperative pain; Visual analog scale (VAS); Intraoperative vital parameters; Recovery quality using the Quality of Recovery-40 (QoR-40) questionnaire; Opioid-related complications	Propofol, ketamine, rocuronium, dexmedetomidine	Propofol, remifentanil, rocuronium
Toleska (16) 2023	Republic of Macedonia	Colorectal surgery	20/20	VAS: the total amount of morphine; the Amount of fentanyl given intravenously; The occurrence of PONV; The total amount of bupivacaine	Dexamethasone, paracetamol, lidocaine, propofol, ketamine, rocuronium bromide, lidocaine, ketamine, magnesium, bupivacaine	Lidocaine, fentanyl, propofol, rocuronium bromide, fentanyl, bupivacaine
Yan (67) 2023	China	Thoracoscopic surgery	80/79	Chronic pain rates, Acute pain rates, Postoperative side effects, Perioperative variables	Dexmedetomidine, propofol, rocuronium, esketamine	Propofol, fentanyl, propofol, ocuronium
Yu (68) 2023	China	Laparoscopic cholecystectomy	75/75	The consumption of rescue analgesics; Time to LMA removal, time to orientation recovery; VAS, PONV, GSS; Time to unassisted walking, sleep quality on the night of surgery, time to first flatus, hemodynamics during induction of general anesthesia	Dexmedetomidine, propofol, lidocaine, cisatracurium, ropivacaine	Propofol, remifentanil, cisatracurium, dexmedetomidine, ropivacaine
Choi (69) 2022	Korea	Gynecological laparoscopy	37/38	Quality of Recovery-40 (QoR-40) questionnaire; Postoperative pain score; Intraoperative and postoperative adverse events; Stress hormones levels	Dexmedetomidine, lidocaine	Remifentanil
An (70) 2022	China	Laparoscopic radical colectomy	51/50	Pain intensity during the operation; Wavelet index, lactic levels, and blood glucose concentration; Visual analog scale (VAS); Rescue analgesic consumption; Side-effects of opioids	Dexmedetomidine, sevofurane, bilateral paravertebral blockade (dexmedetomidine and ropivacaine per side)	Remifentanil, sevofurane, bilateral paravertebral blockade (0.5% ropivacaine per side)
Ibrahim (71) 2022	Saudi Arabia	Sleeve gastrectomy	51/52	Quality of recovery assessed by QoR-40; Postoperative opioid consumption; Time to ambulate; Time to tolerate oral fluid; Time to readiness for discharge	Dexmedetomidine, propofol, ketamine, lidocaine, cisatracurium, OSTAP	Propofol, fentanyl, cisatracurium, OSTAP
Menck (17) 2022	Brazil	Laparoscopic gastroplasty	30/30	VNS; Morphine consumption; Adverse effects of opioids	Magnesium sulfate, ketamine, lidocaine, dexmedetomidine, propofol, rocuronium	Fentanyl, propofol, rocuronium
Saravanaperumal (28) 2022	India	Oocyte retrieval	31/31	Quality of recovery using QOR-15 questionnaire; Bradycardia, post-operative nausea and vomiting, usage of rescue analgesia, and total consumption of propofol	Dexmedetomidine, propofol	Fentanyl, propofol
Tochie (72) 2022	Cameroon	Gynecological surgery	**18/18**	The success rate of OFA, isoflurane consumption, and intraoperative anesthetic complications; Postoperative pain intensity, postoperative complications; Patient satisfaction assessed using the QoR-40 questionnaire, and the financial cost of anesthesia	Magnesium sulfate, lidocaine, ketamine, dexamethasone, propofol, rocuronium, isoflurane, calibrated, ketamine, clonidine	Dexamethasone, diazepam, fentanyl, propofol, rocuronium, isoflurane
Toleska (73) 2022	Republic of Macedonia	Laparoscopic cholecystectomy	40/40	PONV	Dexamethasone, paracetamol, midazolam, lidocaine, propofol, ketamine, rocuronium bromide, magnesium sulfate	Midazolam, fentanyl, propofol, and rocuronium bromide
Van Loocke (74) 2022	Belgium	Laparoscopic bariatric surgery	20/19	Blood glucose level; The total dose of opioids given; The postoperative pain using the VAS (visual analog scale) score; Postoperative nausea and vomiting (PONV), duration of surgery, and surgical and/or anesthetic complications	Dexmedetomidine, lidocaine, esketamine, magnesium	Sufentanil
Beloeil (75) 2021	France	Non-cardiac surgery	157/157	Severe postoperative opioid-related adverse event; Episodes of postoperative pain; Opioid consumption; Postoperative nausea and vomiting	Dexmedetomidine, propofol, desflurane, lidocaine, ketamine, neuromuscular blockade, dexamethasone	Remifentanil, propofol, desflurane, lidocaine, ketamine, neuromuscular blockade, dexamethasone
An (76) 2021	China	Video-assisted thoracoscopic surgery	49/48	Intraoperative PTI; WLI reading, MAP, and HR; Arterial partial pressure of oxygen, blood glucose concentration, and lactic acid value; Total consumption of anesthesia medications; Time to passage of flatus; PONV, length of stay, pH, and SpO_2_	Dexmedetomidine, sevoflurane, thoracic paravertebral blockade, etomidate, cisatracurium, cisatracurium	Remifentanil, sevoflurane, thoracic paravertebral blockade, etomidate, cisatracurium
Taskaldiran (30) 2021	Turkey	Lumbar herniated disc surgery	30/30	Fentanyl consumption and visual analog scale (VAS) score	Propofol, fentanyl, rocuronium, lidocaine, sevoflurane, erector spinae plane block, sugammadex	Propofol, fentanyl, rocuronium, lidocaine, sevoflurane, remifentanil, paracetamol, tramadol, sugammadex
Shah (77) 2020	India	Modified radical mastectomy	35/35	VAS-scores; Hemodynamics; Postoperative complication	Dexmedetomidine, propofol, esmolol, atracurium, paracetamol, PECS blocks, ketamine	Fentanyl, propofol, atracurium, paracetamol, morphine, sevoflurane
Loung (78) 2020	Vietnam	Laparoscopic cholecystectomy	47/47	VAS; Side-effects	Lidocaine, magnesium, ketamine, ketorolac, propofol	Fentanyl, propofol
Hakim (79) 2019	Egypt	Ambulatory gynecologic laparoscopy	40/40	QOR-40 at 24 h postoperative; Postoperative numerical rating scale (NRS); Time to first rescue analgesia; Number of rescue tramadol analgesia; PONV	Dexmedetomidine, propofol, cisatracurium	Fentanyl, propofol, cisatracurium
Toleska (80) 2019	Republic of Macedonia	Laparoscopic cholecystectomy	30/30	Visual Analog Scale (VAS) scores; Opioid requirements	Dexamethasone, paracetamol, midazolam, lidocaine, propofol, rocuronium bromide, ketamine, magnesium sulfate	Midazolam, fentanyl, propofol, and rocuronium bromide
Bhardwaj (59) 2019	India	Laparoscopic urological procedures	40/40	Respiratory depression, mean analgesic consumption, and time to rescue analgesia; Hemodynamic parameters, mean SpO_2_, respiratory rate, and postanesthesia care unit (PACU) discharge time	Dexmedetomidine, propofol, atracurium, lignocaine, ketamine	Fentanyl, propofol, atracurium
Gazi (37) 2018	Turkey	Hysteroscopies	15/15	ANI and VAS; Hemodynamics and complications	Dexmedetomidine, propofol, rocuronium	Remifentanil, propofol, rocuronium
Choi (21) 2017	Korea	Thyroidectomy	40/40	PONV; Pain intensity; Sedation score; Extubation time; Hemodynamics	Dexmedetomidine	Remifentanil
Mogahed (81) 2017	Egypt	Laparoscopic cholecystectomy	40/40	Ramsay Sedation Scale (RSS); Visual Analog Scale (VAS); PONV and the need for additional analgesics or antiemetics	Propofol, rocuronium bromide, sevoflurane, dexmedetomidine	Propofol, rocuronium bromide, sevoflurane, remifentanil
Subasi (82) 2017	Turkey	Laparoscopic cholecystectomy	20/20	Spontaneous respiration, extubation, and response to verbal commands; Aldrete score ≥9 times, postoperative pain scores, and vital parameters; Total analgesic consumption, patients' first analgesic needs	Propofol, rocuronium, fentanyl, dexmedetomidine	Propofol, rocuronium, fentanyl, remifentanil
Hontoir (83) 2016	Belgium	Breast cancer surgery	31/33	QoR-40 score; Postoperative NRS at different timings; Ramsey sedation scale	Clonidine, ketamine, lidocaine, propofol	Remifentanil, ketamine, lidocaine, propofol
Choi (22) 2016	Korea	Laparoscopic hysterectomy	30/30	Pain VAS scores; The modified OAA/S scores, the BIS; Vital signs, and the perioperative side effects	Lidocaine, propofol, rocuronium, N_2_O, desflurane, dexmedetomidine	Lidocaine, propofol, rocuronium, N_2_O, desflurane, remifentanil
Bakan (84) 2015	Turkey	Laparoscopic cholecystectomy	40/40	Postoperative fentanyl consumption; Pain intensity scores (NRS); Incidence of PONV; Other adverse events	Dexmedetomidine, lidocaine, propofol, vecuroniumat	Fentanyl, remifentanil, propofol, lidocaine, vecuroniumat
Hwang (18) 2015	South Korea	Spinal surgery	19/18	Visual analog scale (VAS) score; PCA dosage administered; Postoperative nausea and vomiting (PONV)	Dexmedetomidine, propofol, rocuronium	Remifentanil, propofol, rocuronium
Senol Karataş (23) 2015	Turkey	Major abdominal surgery	16/16	Meperidine consumption, VAS scores, Side effects	Atropine sulfate, midazolam, thiopental sodium, vecuronium, desflurane, paracetamol	Atropine sulfate, midazolam, thiopental sodium, vecuronium, desflurane, paracetamol, remifentanil
White (85) 2015	America	Superficial surgical	50/50	Number of coughing episodes; Vital signs; Dosages of all anesthetic drugs; Duration of surgery and anesthesia; VRS scores and side effects	Lidocaine, propofol, desflurane	Fentanyl, lidocaine, propofol, desflurane
Sahoo (19) 2015	India	Laparoscopic gynecological surgeries	80/80	pain intensity, analgesic requirements, and postoperative nausea and vomiting (PONV)	Dexmedetomidine, vercuronium, propofol	Remifentanyl, propofol, vercuronium
Mansour (20) 2013	Egypt	Bariatric surgery	15/13	Hemodynamics; Pain monitoring (VAS); Post-operative nausea and vomiting; Patient satisfaction and acute pain nurse satisfaction	Propofol, ketamine, rocuronium, sevoflurane	Propofol, fentanyl, rocuronium, sevoflurane
Lee (86) 2013	Korea	Endoscopic sinus surgery	32/34	Surgical conditions, hemodynamic parameters, intraoperative blood loss, time to extubation, sedation, and pain	Propofol, desflurane, dexmedetomidine	Propofol, desflurane, remifentanil
Techanivate (87) 2012	Thailand	Gynecologic laparoscopy	20/20	Pain intensity using verbal rating score (VRS); The severity of sedation; The episode of intraoperative and postoperative side effects	Propofol, atracurium, desflurane, dexmedetomidine	Propofol, atracurium, desflurane, nitrous oxide, fentanyl
Lee (31) 2012	Korea	Laparoscopic hysterectomy	25/28	Pain score, Total volume of administered patient-controlled analgesia (PCA), and PONV	Glycopyrrolate, midazolam, thiopental sodium, rocuronium, desflurane, nitrous oxide	Glycopyrrolate, midazolam, thiopental sodium, rocuronium, desflurane, nitrous oxide, sufentanil
Lee (29) 2011	Korea	Tonsillectomy	30/30	Degree of pain severity, First postoperative requirement, Analgesic dose required	Sevoflurane	Sovoflurane, remifentanil
Jung (24) 2011	Korea	Laparoscopic hysterectomy	25/25	Visual analog scale (VAS) scores; Alertness (OAA/S) score; Postoperative side-effects; BIS, VAS scores, modified OAA/S scores of sedation, vital signs, respiratory rates, and end-tidal carbon dioxide levels	Lidocaine, propofol, rocuronium, nitrous oxide, desflurane, dexmedetomidine	Lidocaine, propofol, rocuronium, nitrous oxide, desflurane, remifentanil
De (26) 2010	France	Minor hand surgery	30/30	Postoperative pain; Postoperative vomiting; Time to discharge from the recovery room; Time to discharge home	Midazolam, sevoflurane, mix of O_2_/N_2_O (33%/66%), by wrist blocks	Alfentanil, propacetamol, niflumic acid
Ryu (88) 2009	Korea	Middle ear surgery	40/40	Haemodynamic variables, surgical conditions, postoperative pain, and adverse effects	Propofol, sevoflurane, magnesium sulfate	Propofol, sevoflurane, remifentanil
Salman (89) 2009	Turkey	Gynecologic laparoscopic surgery	30/30	Demographic, hemodynamic data, postoperative pain scores, and discharge time; Time to extubation, to orientation to person, to place, and date; Postoperative nausea, vomiting, and analgesic requirements	Propofol, vecuronium bromide, dexmedetomidine	Propofol, vecuronium bromide, remifentanil
Collard (90) 2007	Canada	Cholecystectomy	30/28	VRS; PONV; Itching, urinary retention; White-song score	Midazolam, remifentanil or fentanyl, propofol, rocuronium	Midazolam, esmolol, propofol, rocuronium
Feld (91) 2006	America	Bariatric surgery	10/10	Patient-evaluated pain scores; Morphine use by patient-controlled analgesia pump	Dexmedetomidine, midazolam, lidocaine, thiopental, succinylcholine	Fentanyl, lidocaine, thiopental, succinylcholine
Shirakami (92) 2006	Japan	Major breast cancer surgery	26/25	Postoperative nausea and vomiting and postanesthesia recovery	Propofol, lidocaine, diclofenac sodium, local infiltration anesthesia (0.5% lidocaine, 200 mg × 2; total, 400 mg)	Propofol, lidocaine, diclofenac sodium, local infiltration anesthesia (0.5% lidocaine, 200 mg × 2; total, 400 mg), fentanyl
Feld (91) 2005	America	Bariatric surgery	10/10	Patient-evaluated pain scores; Morphine use by patient-controlled analgesia pump	Midazolam, dexmedetomidine, lidocaine, thiopental, succinylcholine	Fentanyl, lidocaine, thiopental, succinylcholine, desflurane
Hansen (32) 2005	Denmark	Major abdominal surgery	18/21	Patient-controlled analgesia with morphine for 24 h post-operatively; Morphine consumption; Assessment of pain; Side-effects and levels of sensory block	Triazolam, thoracic epidural anesthesia, thiopental, rocuronium, sevoflurane	Triazolam, thoracic epidural anesthesia, thiopental, rocuronium, sevoflurane, remifentanil
Feld (33) 2003	America	Gastric bypass surgery	15/15	Visual analog scale; Morphine use by patient-controlled analgesia (PCA) pump	Ventilated, sevoflurane, ketorolac, clonidine, lidocaine, ketamine, magnesium sulfate, methylprednisolone	Sevoflurane, fentanyl
Curry (25) 1996	America	Electrocautery tubal ligation	22/22	Patient assessments of visual analog scales (VAS); Cumulative opioid requirements.	Propofol, lidocaine, vecuronium, nitrous oxide, isoflurane, neostigmine, glycopyrrolate	Propofol, lidocaine, vecuronium, nitrous oxide, isoflurane, neostigmine, glycopyrrolate, fentanyl
Katz (93) 1996	Canada	Abdominalhysterectomy	15/15	Pain and morphine consumption; Plasma levels of aljentanil; Adverse effects	Midazolam, thiopentone, isoflurane, N_2_O, vecuronium	Midazolam, aIfentanil, N_2_O, vecuronium
Sukhani (94) 1996	America	Ambulatory gynecologic laparoscopy	39/38	Post-analgesic effect; sequelae of vomiting; recovery characteristics (awakening, Walking, and discharge)	Lidocaine, propofol, atracurium, nitrous oxide, ketorolac	Lidocaine, propofol, atracurium, nitrous oxide, fentanyl
Tverskoy (34) 1994	Israel	Lective hysterectomy	9/9	VAS	Ketamine, thiopental, isoflurane	Fentanyl, thiopental, isoflurane

OFA, Opioid-Free Anesthesia; OBA, Opioid-Based Anesthesia; PONV, Postoperative Nausea and Vomiting; LOS, length of stay in hospital; VAS, Visual Analog Scale; NRS, Numeric Rating Scale; QoR-40, Quality of Recovery-40; OSTAP, Oblique Subcostal Transversus Abdominis Plane block; QLB, Quadratus Lumborum Block; TAP, Transversus Abdominis Plane block; D-SAPB, Deep Serratus Anterior Plane Block.

### 3.2 Risk of bias assessment of the included studies

**Figure 2:**
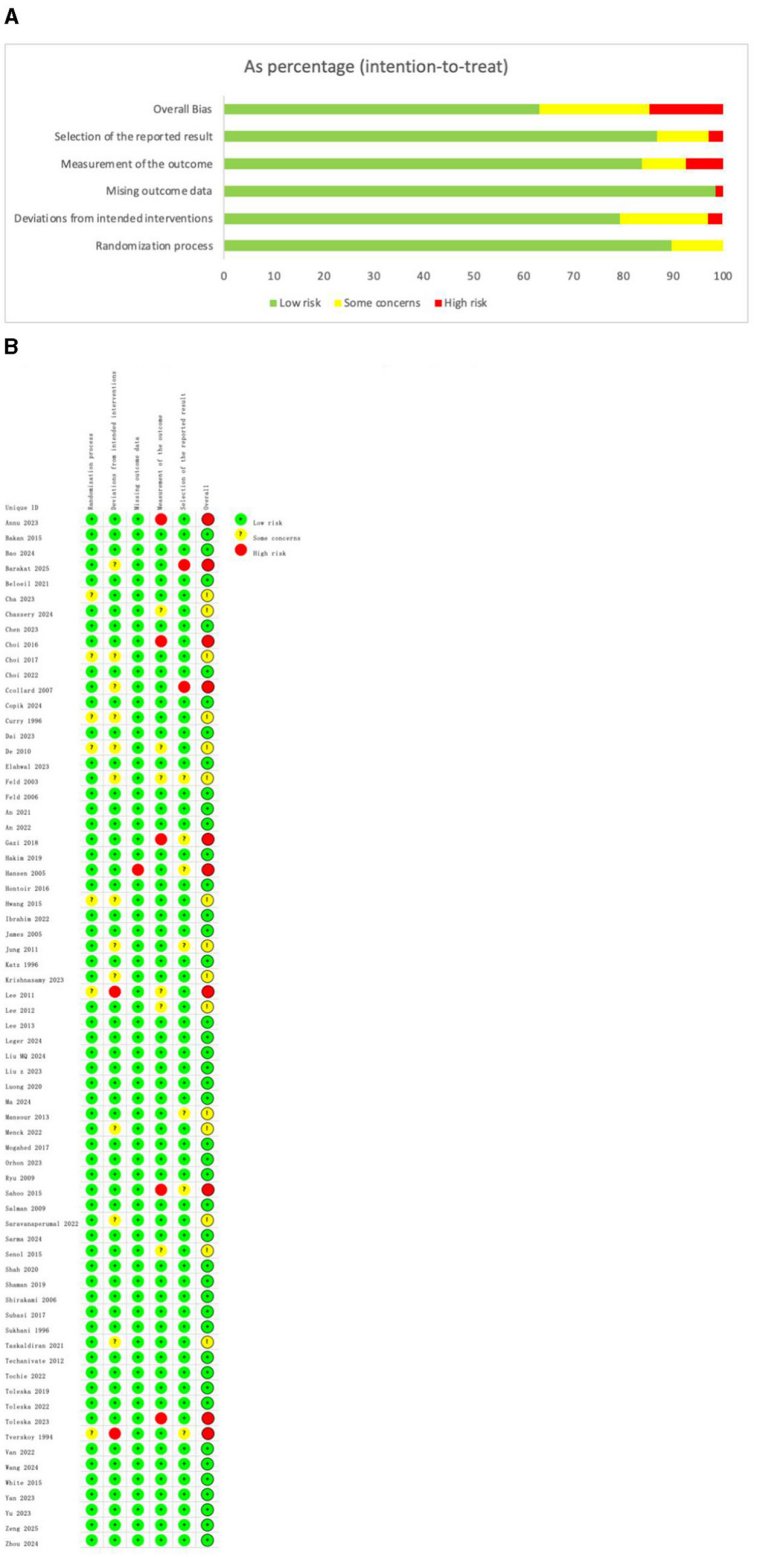
**(A)** The proportion of each methodological quality item. **(B)** The methodological quality assessment.

Overall, 43 trials were classified as having a low risk of bias, 15 trials as having an unclear risk of bias, and 10 trials as having a high risk of bias. Among the 68 trials, randomization procedures were fully described in 55 trials (81%), and the concealment of treatment allocation was described in 52 trials (76%). One study had unclear or high-risk incomplete outcome data. The evaluation of study quality using the RoB 2 tool is provided in Supplementary Table 3. The proportion of each methodological quality item is shown in Figure 2A, and the methodological quality assessment of the included studies is shown in Figure 2B.

### 3.3 Meta-analysis results

#### 3.3.1 Primary outcome indicator

##### 3.3.1.1 PONV

**Figure 3:**
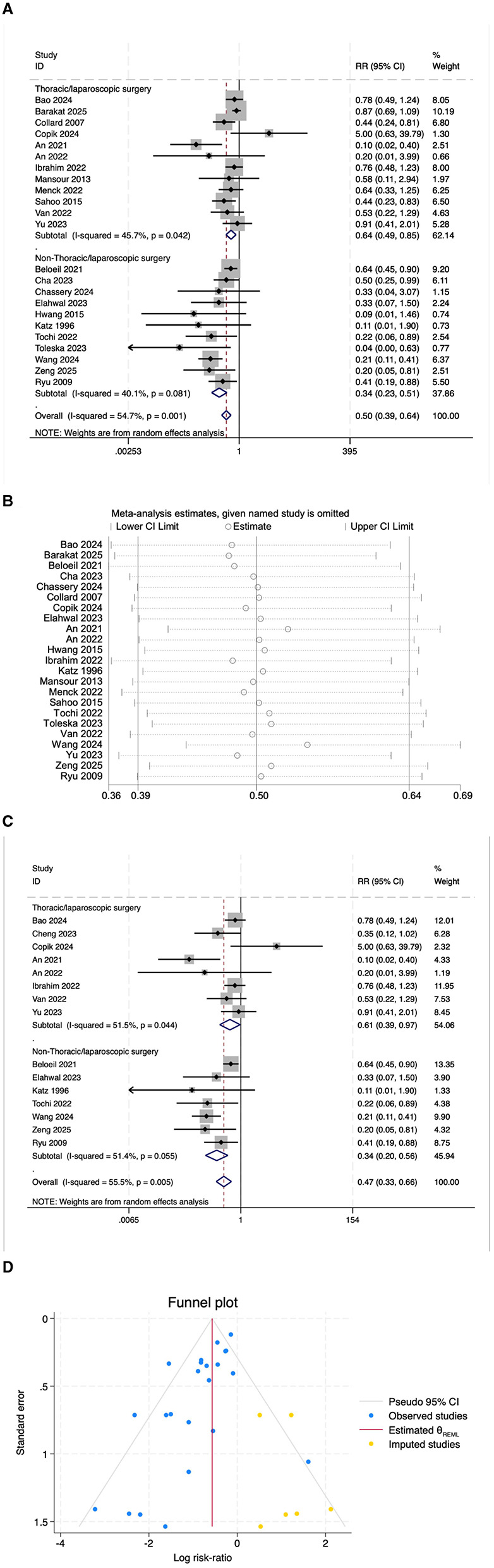
**(A)** The forest plot of the impact of OFA on PONV. **(B)** The sensitivity analysis plot of the impact of OFA on PONV. **(C)** The forest plot of the impact of OFA on PONV after excluding low-quality studies. **(D)** Trim and fill method for PONV.

A total of 23 studies reported on the incidence of postoperative PONV. The results indicated that the risk of PONV was significantly lower in the OFA group compared with the OBA group (RR = 0.50, 95% CI: 0.39–0.64, *I*^2^ = 54.7%, *P*_*h*_ = 0.001, Figure 3A). Subgroup analyses revealed significant reductions in PONV risk in both the thoracoscopic/laparoscopic surgery group (RR = 0.64, 95% CI: 0.49–0.85, I^2^ = 45.7%, P_h_ = 0.042, Figure 3A) and the non-thoracoscopic/laparoscopic surgery group (RR = 0.34, 95% CI: 0.23–0.51, I^2^ = 40.1%, P_h_ = 0.081, Figure 3A). Additional subgroup analyses are presented in Table 2. Sensitivity analysis was initially performed using a stepwise exclusion method, with consistent results (Figure 3B). After excluding low-quality studies (14–20), the combined effect size remained stable (RR = 0.47, 95% CI: 0.33–0.66, *I*^2^ = 55.5%, *P*_*h*_ = 0.005, Figure 3C). The funnel plot distribution and the result of Egger's test (*P* = 0.002) suggested the presence of publication bias. Adjustment using the trim and fill method (Figure 3D) maintained a robust pooled effect (RR = 0.566, 95% CI: 0.442–0.726) without changing the overall direction of the result. The GRADE assessment for PONV is shown in Supplementary Figure 1.

**Table 2:** Subgroup meta-analysis and meta-regression analysis of OFA outcomes by surgical type, anesthetic technique, and use of regional nerve block.

**Outcome**	**Grouping method**	**No. of studies**	**Results of meta-analysis**			**The meta-regression analysis results of the total combined analysis**			
			**RR (95% CI)**	***I*** ^2^ **(%)**	***P***_h_	**Coefficient (95% CI)**	***SE***	***t***	***P***
**PONV**									
Overall		23	0.50 (0.39–0.64)	54.7	0.001	NR	NR	NR	NR
Surgical type	Thoracic/laparoscopic surgery	12	0.64 (0.49–0.85)	45.7	0.042	−0.624 (−1.187−0.061)	0.269	−2.32	**0.032**
	Non-Thoracic/laparoscopic surgery	11	0.34 (0.23–0.51)	40.1	0.081				
Anesthetic technique	TIVA	14	0.53 (0.39–0.72)	47.9	0.023	0.079 (−0.501–0.659)	0.277	0.289	0.779
	Intravenous inhalational anesthesia	9	0.44 (0.27–0.69)	61.9	0.007				
Regional nerve block	Yes	4	0.50 (0.27–0.93)	62.6	0.045	0.140 (−0.515–0.795)	0.313	0.447	0.660
	No	19	0.49 (0.36–0.66)	55.6	0.002				
**Nausea**									
Overall		22	0.34 (0.25–0.46)	27.5	0.115	NR	NR	NR	NR
Surgical type	Thoracic/laparoscopic surgery	12	0.29 (0.20–0.42)	0.8	0.436	0.238 (−0.347–0.823)	0.278	0.85	0.404
	Non-Thoracic/laparoscopic surgery	10	0.40 (0.24–0.64)	44.9	0.060				
Anesthetic technique	TIVA	14	0.66 (0.36–1.21)	28.7	0.199	0.809 (0.162–1.455)	0.308	2.63	**0.017**
	Intravenous inhalational anesthesia	8	0.28 (0.21–0.37)	0.0	0.755				
Regional nerve block	Yes	2	0.21 (0.08–0.53)	0.0	0.525	−0.444 (−1.554–0.666)	0.528	−0.84	0.412
	No	20	0.36 (0.26–0.49)	30.2	0.100				
**Vomiting**									
Overall		23	0.34 (0.25–0.46)	0.0	0.596	NR	NR	NR	NR
Surgical type	Thoracic/laparoscopic surgery	14	0.33 (0.23–0.48)	8.0	0.365	−0.107 (−0.991–0.776)	0.422	−0.25	0.802
	Non-Thoracic/laparoscopic surgery	9	0.28 (0.13–0.60)	0.0	0.715				
Anesthetic technique	TIVA	13	0.19 (0.11–0.30)	0.0	0.999	1.073 (0.333–1.814)	0.354	3.03	**0.007**
	Intravenous inhalational anesthesia	10	0.50 (0.34–0.74)	0.0	0.574				
Regional nerve block	Yes	3	0.42 (0.24–0.76)	0.0	0.441	−0.212 (−1.026–0.602)	0.389	−0.54	0.592
	No	20	0.31 (0.22–0.45)	0.0	0.563				
**Postoperative emergency analgesia needs**									
Overall		26	0.61 (0.51–0.72)	48.8	0.003	NR	NR	NR	NR
Surgical type	Thoracic/laparoscopic surgery	15	0.61 (0.50–0.75)	43.5	0.037	0.048 (−0.378–0.473)	0.205	0.23	0.819
	Non-Thoracic/laparoscopic surgery	11	0.54 (0.38–0.78)	56.5	0.011				
Anesthetic technique	TIVA	16	0.57 (0.45–0.73)	52.5	0.007	0.143 (−0.249–0.534)	0.189	0.76	0.458
	Intravenous inhalational anesthesia	10	0.65 (0.51–0.84)	44.9	0.060				
Regional nerve block	Yes	3	0.80 (0.60–1.07)	10.5	0.327	0.397 (−0.181–0.975)	0.279	1.42	0.169
	No	23	0.57 (0.47–0.70)	49.3	0.004				
**LOS**									
Overall		9	−0.06 (−0.18–0.06)	26.5	0.208	NR	NR	NR	NR
Surgical type	Thoracic/laparoscopic surgery	5	0.04 (−0.15–0.22)	36.5	0.178	−0.278 (−0.668–0.112)	0.152	−1.83	0.127
	Non-Thoracic/laparoscopic surgery	4	−0.12 (−0.28–0.02)	0.0	0.413				
Anesthetic technique	TIVA	4	−0.09 (−0.26–0.09)	51.8	0.101	−0.157 (−0.639–0.325)	0.188	−0.84	0.441
	Intravenous inhalational anesthesia	5	−0.04 (−0.19–0.12)	10.7	0.345				
Regional nerve block	Yes	5	−0.02 (−0.16–0.13)	0.0	0.424	0.345 (−0.189–0.879)	0.208	1.66	0.158
	No	4	−0.14 (−0.34–0.06)	50.3	0.110				
**Postoperative respiratory dysfunction**									
Overall		9	0.29 (0.09–0.91)	68.5	0.001	NR	NR	NR	NR
Surgical type	Thoracic/laparoscopic surgery	7	0.47 (0.15–1.46)	50.8	0.058	1.215 (−2.310–4.739)	1.371	0.89	0.416
	Non-Thoracic/laparoscopic surgery	2	0.10 (0.02–0.57)	30.5	0.230				
Anesthetic technique	TIVA	7	0.21 (0.07–0.61)	26.5	0.227	0.434 (−3.420–4.288)	1.499	0.29	0.784
	Intravenous inhalational anesthesia	2	0.69 (0.11–4.12)	50.1	0.157				
Regional nerve block	Yes	2	0.69 (0.12–4.12)	49.4	0.160	0.455 (−3.410–4.300)	1.499	0.30	0.779
	No	7	0.21 (0.07–0.61)	26.5	0.227				
**Bradycardia**									
Overall		14	1.04 (0.63–1.70)	42.1	0.048	NR	NR	NR	NR
Surgical type	Thoracic/laparoscopic surgery	7	1.10 (0.62–1.96)	52.3	0.050	−0.121 (−1.696–1.454)	0.707	−0.17	0.867
	Non-Thoracic/laparoscopic surgery	7	0.87 (0.30–2.53)	38.2	0.137				
Anesthetic technique	TIVA	9	0.91 (0.49–1.69)	42.6	0.083	0.127 (−1.403–1.658)	0.687	0.19	0.857
	Intravenous inhalational anesthesia	5	1.39 (0.59–3.25)	20.4	0.285				
Regional nerve block	Yes	3	1.55 (0.72–3.31)	65.8	0.054	0.820 (−0.799–2.440)	0.727	1.13	0.285
	No	11	0.77 (0.39–1.53)	33.2	0.133				
**Postoperative intestinal dysfunction**									
Overall		5	0.25 (0.14–0.46)	0.0	0.624	NR	NR	NR	NR
Anesthetic technique	TIVA	3	0.27 (0.14–0.54)	0.0	0.826	−0.577 (−4.890–3.736)	1.002	−0.58	0.623
	Intravenous inhalational anesthesia	2	0.18 (0.03–1.03)	50.5	0.155				
Regional nerve block	Yes	1	0.24 (0.10–0.56)	NR	NR	−0.341 (−4.480–3.798)	0.962	−0.35	0.757
	No	4	0.26 (0.11–0.61)	0.0	0.460				

RR, relative risk; CI, confidence interval; I^2^, heterogeneity statistic; Ph, *p*-value for heterogeneity test; Coefficient (95% CI), the regression coefficient and its 95% confidence interval; SE, Standard error; *t, t*-test value; *p, p*-value, with values < 0.05 indicating statistical significance; NR, not reported.

Data are grouped by: anesthesia technique (TIVA, intravenous inhalational anesthesia) and regional nerve block status (yes/no).

PONV, Postoperative Nausea and Vomiting; LOS, Length of Stay.

Since all cases of Postoperative intestinal dysfunction were non-thoracoscopic and non-laparoscopic surgeries, subgroup analysis by surgical approach was not conducted. The coefficients in this table were derived by incorporating all three factors (Surgical Type, Anesthetic Technique, and Regional Nerve Block) simultaneously into the meta-regression model, rather than analyzing each factor individually. This approach reflects the impact on the incidence of PONV when considering these factors collectively.

Bold values in the table indicate *p*-values < 0.05, which are statistically significant.

##### 3.3.1.2 Nausea and vomiting

**Figure 4:**
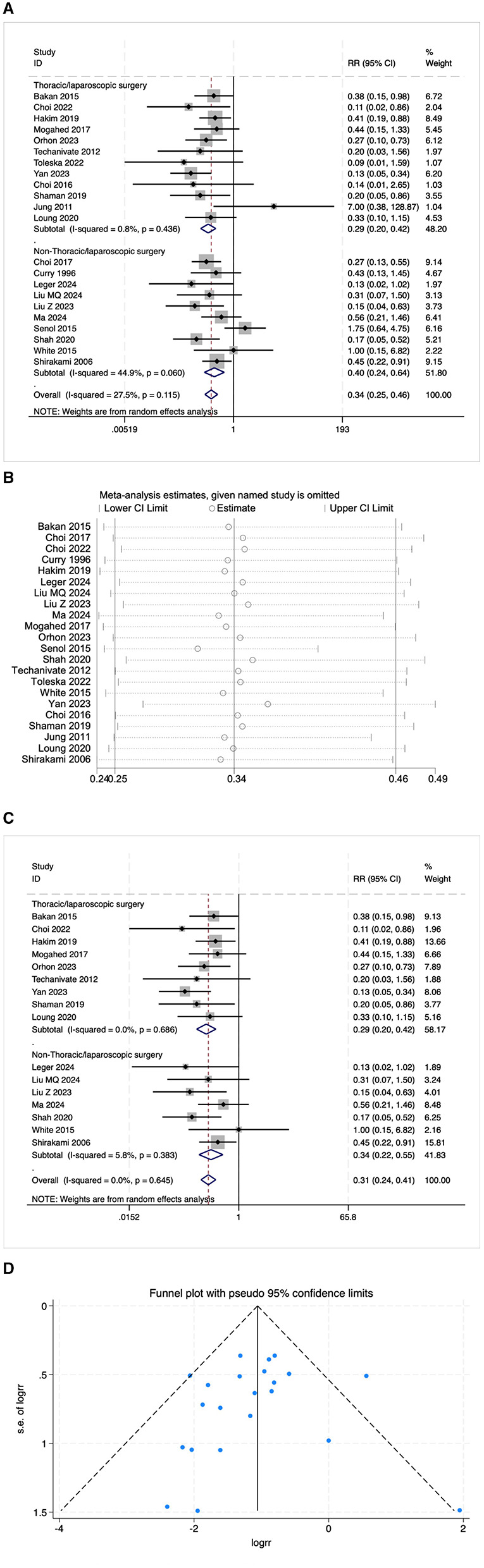
**(A)** The forest plot of the impact of OFA on nausea. **(B)** The sensitivity analysis plot of the impact of OFA on nausea. **(C)** The forest plot of the impact of OFA on nausea after excluding low-quality studies. **(D)** The funnel plot of the impact of OFA on nausea.

Some studies reported PONV as postoperative nausea and postoperative vomiting, respectively. This review included 22 studies on postoperative nausea and 23 studies on postoperative vomiting. The results indicated that the risk of postoperative nausea in the OFA group was lower than that in the OBA group (RR = 0.34, 95% CI: 0.25–0.46, *I*^2^ = 27.5%, *P*_*h*_ = 0.115, Figure 4A). The results of subgroup analysis showed that both the thoracoscopic/laparoscopic surgery group (RR = 0.29, 95% CI: 0.20–0.42, *I*^2^ = 0.8%, *P*_*h*_ = 0.436, Figure 4A) and the non-thoracoscopic/laparoscopic surgery group (RR = 0.40, 95% CI: 0.24–0.64, *I*^2^ = 44.9%, *P*_*h*_ = 0.060, Figure 4A) demonstrated a significant risk reduction. Additional subgroup analyses are detailed in Table 2. The elimination method was applied step-by-step, and the results were consistent (Figure 4B). After excluding low-quality studies (16, 21–25), the combined effect size remained stable (RR = 0.31, 95% CI: 0.24–0.41, *I*^2^ = 0.00%, *P*_*h*_ = 0.645, Figure 4C). The distribution of the funnel plot (Figure 4D) and the result of Egger's test (*P* = 0.543) also showed no significant publication bias.

**Figure 5:**
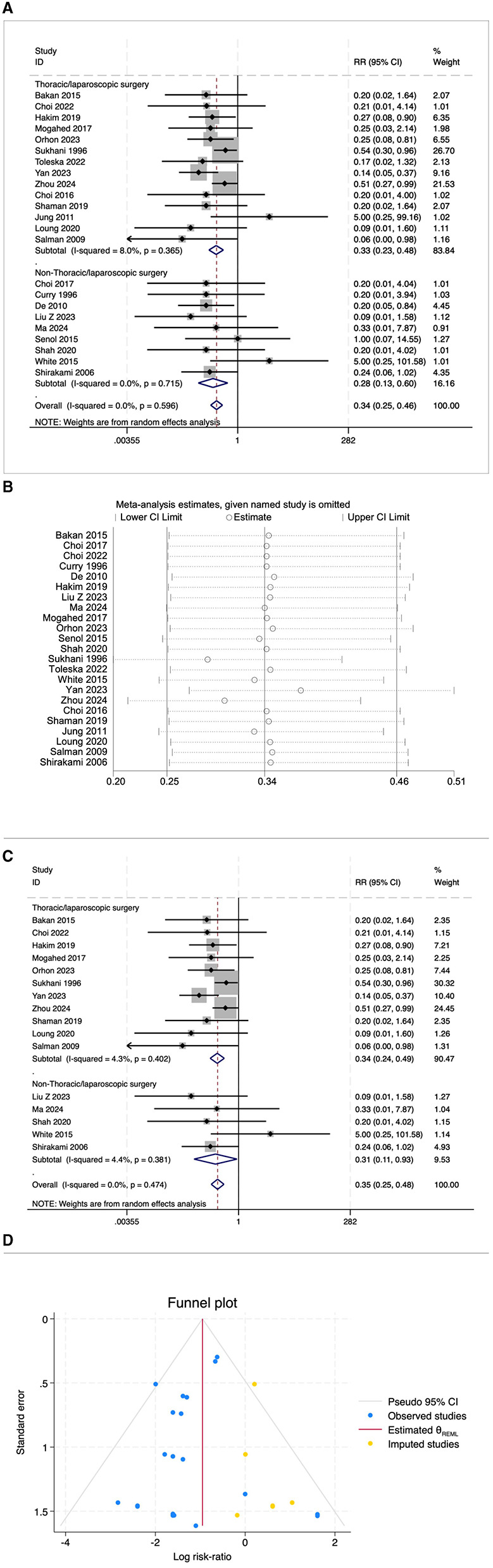
**(A)** The forest plot of the impact of OFA on vomiting. **(B)** The sensitivity analysis plot of the impact of OFA on vomiting. **(C)** The forest plot of the impact of OFA on vomiting after excluding low-quality studies. **(D)** Trim and fill method for vomiting.

The risk of postoperative vomiting in the OFA group was also lower than that in the OBA group (RR = 0.34, 95% CI: 0.25–0.46, *I*^2^ = 0.0%, *P*_*h*_ = 0.596, Figure 5A). The results of subgroup analysis showed that both the thoracoscopic/laparoscopic surgery group (RR = 0.33, 95% CI: 0.23–0.48, *I*^2^ = 8.0%, *P*_*h*_ = 0.365, Figure 5A) and the non-thoracoscopic/laparoscopic surgery group (RR = 0.28, 95% CI: 0.13–0.60, *I*^2^ = 0.00%, *P*_*h*_ = 0.715, Figure 5A) demonstrated a significant risk reduction. Additional subgroup analyses are detailed in Table 2. The elimination method was applied step-by-step, and the results were consistent (Figure 5B). After excluding low-quality studies (16, 21–26), the combined effect size remained stable (RR = 0.35, 95% CI: 0.25–0.48, *I*^2^ = 0.0%, *P*_*h*_ = 0.474, Figure 5C). The result of Egger's test (*P* = 0.156) indicated the presence of publication bias, but the distribution of the funnel plot appears asymmetrical when using the trim and fill method for adjustment (Figure 5D). The pooled effect remained robust (RR = 0.39, 95% CI: 0.27–0.55) without changing the direction of the result. The GRADE assessment for nausea and vomiting is shown in Supplementary Figure 1.

#### 3.3.2 Secondary outcome indicators

The perioperative recovery quality indicators in this study include: postoperative analgesic demand (whether emergency analgesia is needed); length of stay in hospital (LOS); postoperative adverse reactions; pain scores; postoperative quality of recovery (QoR-40) score; extubation time; duration of stay in PACU.

##### 3.3.2.1 Postoperative emergency analgesia needs

**Figure 6:**
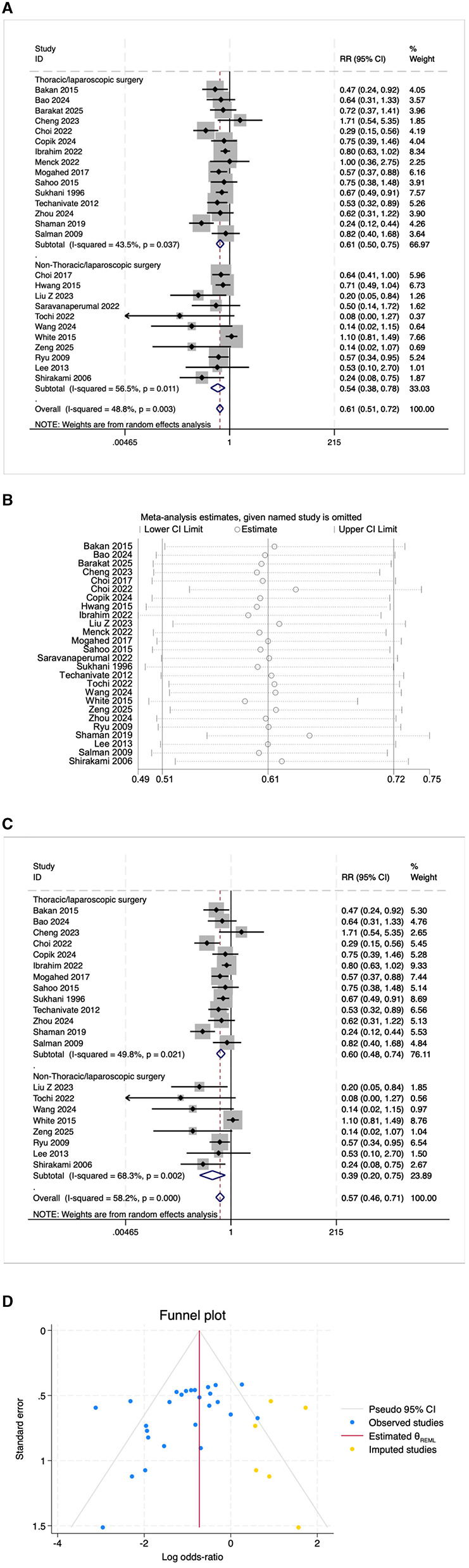
**(A)** The forest plot of the impact of OFA on postoperative emergency analgesia needs. **(B)** The sensitivity analysis plot of the impact of OFA on postoperative emergency analgesia needs. **(C)** The forest plot of the impact of OFA on postoperative emergency analgesia needs after excluding low-quality studies. **(D)** Trim and fill method for postoperative emergency analgesia needs.

Twenty-six RCTS reported the need for postoperative emergency analgesia. The results indicated that the number of patients in the OFA group requiring emergency analgesia after surgery was significantly lower than that in the OBA group (RR = 0.61, 95% CI: 0.51–0.72, *I*^2^ = 48.8%, *P*_*h*_ = 0.003, Figure 6A). The results of subgroup analysis indicated that both the thoracoscopic/laparoscopic surgery group (RR = 0.61, 95% CI: 0.50–0.75, *I*^2^ = 43.5%, *P*_*h*_ = 0.037, Figure 6A) and the non-thoracoscopic/laparoscopic surgery group (RR = 0.54, 95% CI: 0.38–0.78, *I*^2^ = 56.5%, *P*_*h*_ = 0.011, Figure 6A) demonstrated a significant risk reduction. Additional subgroup analyses are detailed in Table 2. The elimination method was applied step-by-step, and the results were consistent (Figure 6B). After excluding low-quality studies (17, 18, 21, 27, 28), the combined effect size remained stable (RR = 0.57, 95% CI: 0.46–0.71, Figure 6C). The distribution of the funnel plot and the result of Egger's test (*P* = 0.005) indicated the presence of publication bias. The combined result of the trim and fill method (Figure 6D) was RR = 0.484 (95% CI: 0.330–0.710), without reversal. The GRADE assessment for postoperative emergency analgesia needs is presented in Supplementary Figure 1.

##### 3.3.2.2 LOS

**Figure 7:**
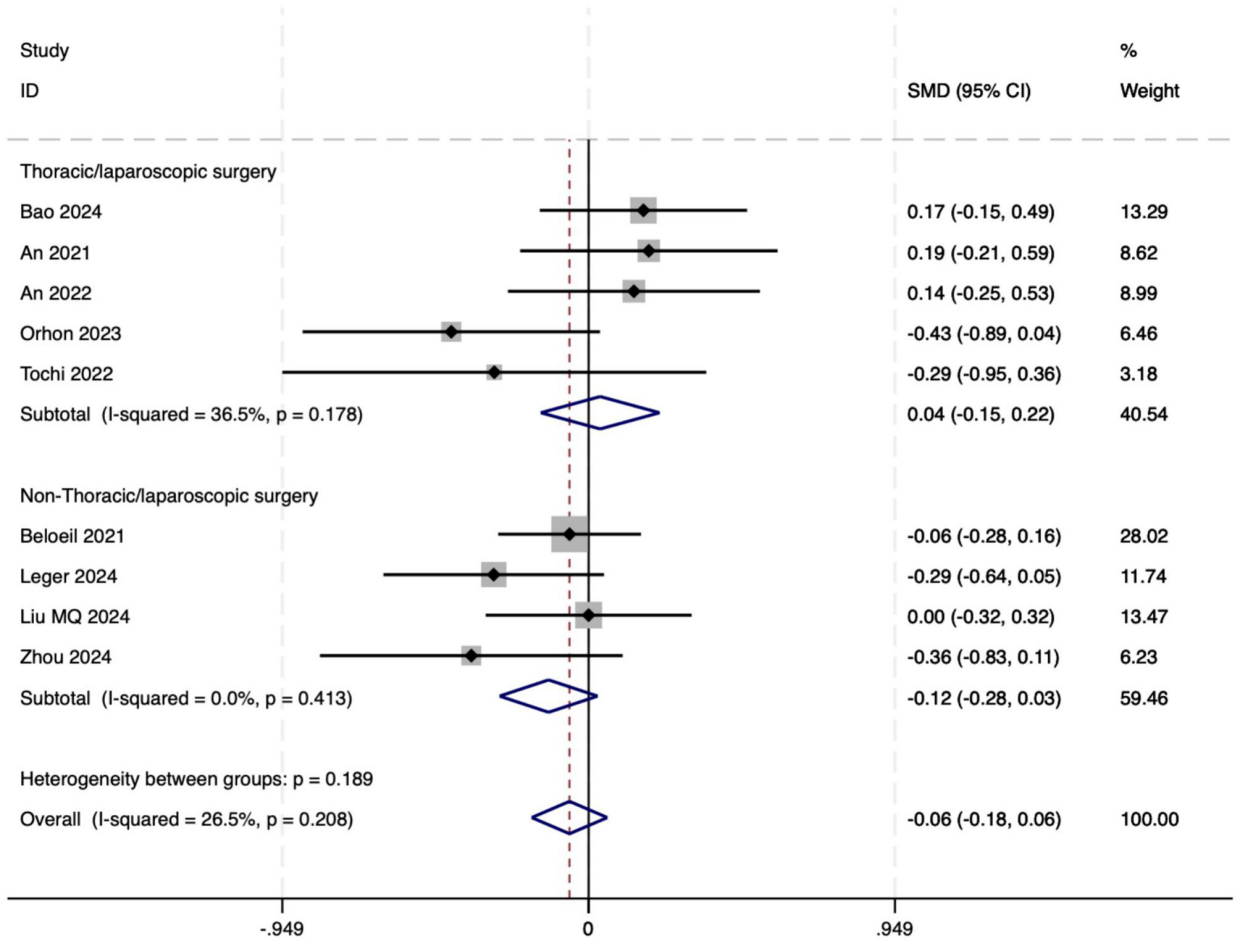
The forest plot of the impact of OFA on LOS.

The LOS was evaluated in nine trials. There was no significant difference in the length of hospital stay between the OFA group and the OBA group (SMD = −0.06, 95% CI: −0.18–0.06, *I*^2^ = 26.5%, *P*_*h*_ = 0.208, Figure 7). Further subgroup analysis was conducted in the thoracoscopic/laparoscopic surgery group (SMD = 0.04, 95% CI: −0.15–0.22, *I*^2^ = 36.5%, *P*_*h*_ = 0.178, Figure 7) and the non-thoracoscopic/laparoscopic surgery group (SMD = −0.12, 95% CI: −0.28–0.03, *I*^2^ = 0.00%, *P*_*h*_ = 0.413, Figure 7), both of which showed stability. Additional subgroup analyses are detailed in Table 2. The GRADE assessment for LOS is shown in Supplementary Figure 1.

##### 3.3.2.3 Postoperative adverse reactions

**Figure 8:**
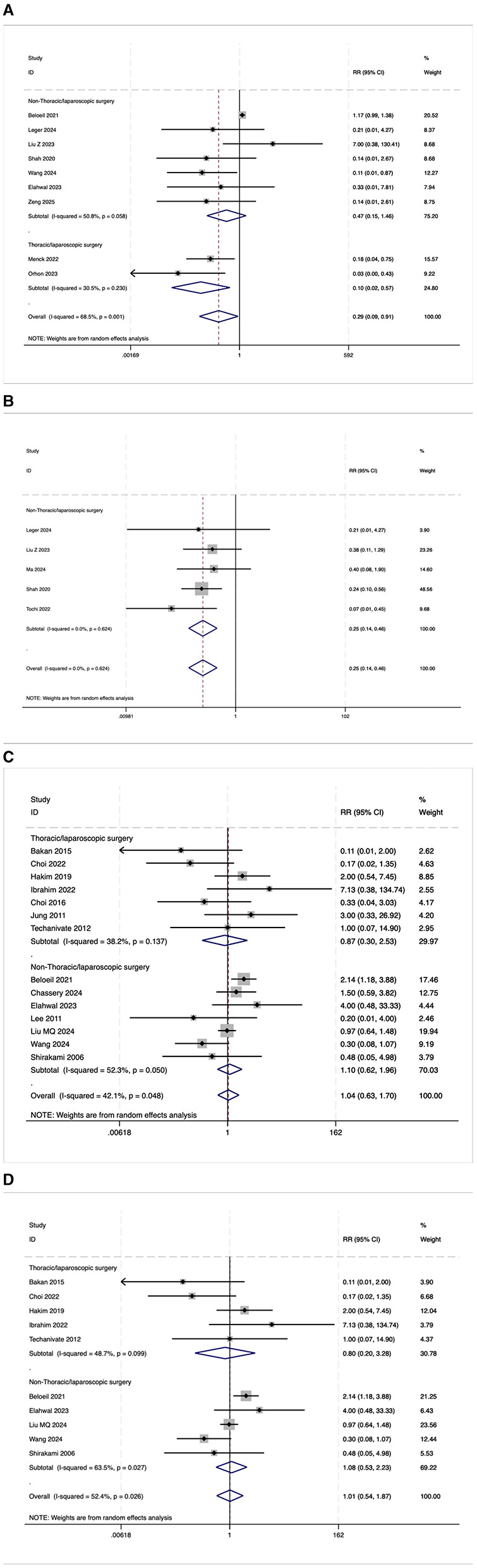
**(A)** The forest plot of the impact of OFA on postoperative respiratory dysfunction. **(B)** The forest plot of the impact of OFA on postoperative intestinal dysfunction. **(C)** The forest plot of the impact of OFA on bradycardia. **(D)** The forest plot of the impact of OFA on bradycardia after excluding low-quality studies.

Nine studies reported the incidence of postoperative respiratory dysfunction. The results indicated that the incidence in the OFA group was lower than that in the OBA group (RR = 0.29, 95% CI: 0.09–0.91, *I*^2^ = 68.5%, *P*_*h*_ = 0.001, Figure 8A). Five studies reported postoperative intestinal dysfunction. The results indicated that the incidence of postoperative intestinal dysfunction in the OFA group was lower than that in the OBA group (RR = 0.25, 95% CI: 0.14–0.46, *I*^2^ = 0.00%, *P*_*h*_ = 0.624, Figure 8B). Fourteen studies reported bradycardia. The results indicated that there was no significant difference in the incidence of bradycardia during the operation between the OFA group and the OBA group (RR = 1.04, 95% CI: 0.63–1.70, *I*^2^ = 42.1%, *P*_*h*_ = 0.048, Figure 8C), after excluding low-quality studies (14, 22, 24, 29) and the combined effect size remained stable (RR = 1.01, 95% CI: 0.54–1.87, *I*^2^ = 52.4%, *P*_*h*_ = 0.026, Figure 8D). Additional subgroup analyses are detailed in Table 2. The GRADE assessment for postoperative adverse reactions is depicted in Supplementary Figure 1.

##### 3.3.2.4 NRS scores

**Figure 9:**
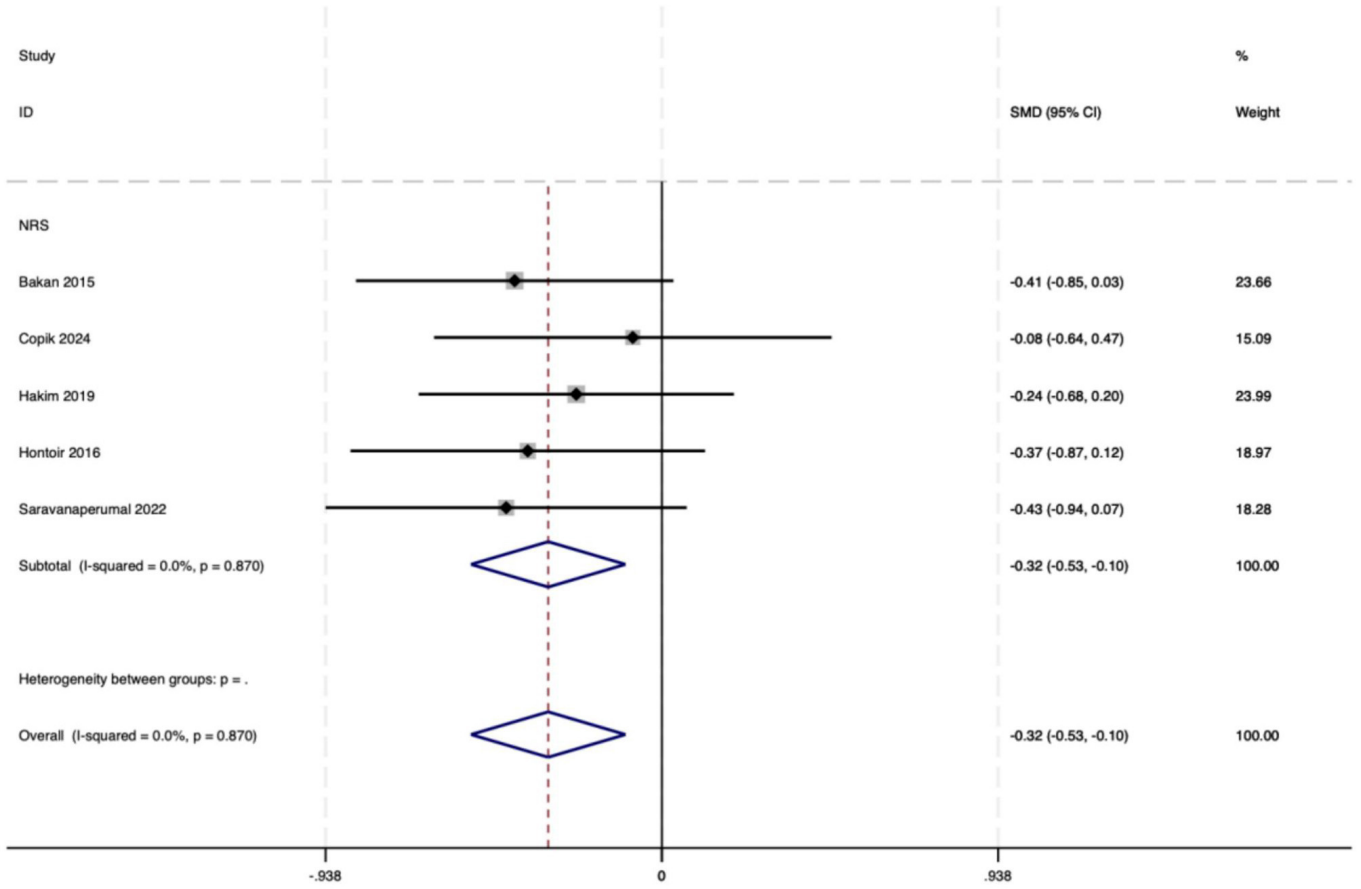
The forest plot of the impact of OFA on NRS.

The pain score was calculated using NRS or VAS at 24 h after the operation. Five studies statistically analyzed the NRS scores 24 h after the operation. The results showed that the NRS pain score in the OFA group was lower than that in the OBA group (SMD = −0.32, 95% CI: −0.53 to −0.10, *I*^2^ = 0.00%, *P*_*h*_ = 0.870, Figure 9). The GRADE assessment for NRS is shown in Supplementary Figure 1.

### 3.4 Meta-regression analysis

Meta-regression was performed to evaluate the influence of surgical type, anesthetic technique, and the use of nerve block on postoperative outcomes and to identify potential sources of heterogeneity. The analysis revealed that both surgical type and anesthetic technique significantly affected certain postoperative outcomes. Specifically, thoracic/laparoscopic surgery (compared with non-thoracic/laparoscopic surgery) was associated with a higher risk of PONV (coefficient = −0.624, *P* = 0.032, Table 2). However, surgical type did not significantly influence the need for postoperative emergency analgesia, LOS, or postoperative respiratory dysfunction, suggesting that heterogeneity in these outcomes may not be primarily attributable to surgical type.

Additionally, the use of intravenous inhalational anesthesia (compared with TIVA) was associated with a higher risk of nausea (coefficient = 0.809, *P* = 0.017, Table 2) and vomiting (coefficient = 1.073, *P* = 0.007, Table 2). In contrast, anesthetic technique did not significantly impact the need for postoperative emergency analgesia, LOS, or postoperative respiratory dysfunction, suggesting that other factors may be more influential in these cases. The use of nerve block did not show significant associations with any of the examined outcomes. Overall, these results emphasize the importance of considering surgical type and anesthetic technique when evaluating postoperative outcomes, as they may significantly influence specific adverse events such as PONV, nausea, and vomiting.

### 3.5 Results of systematic review

#### 3.5.1 VAS scores

**Figure 10:**
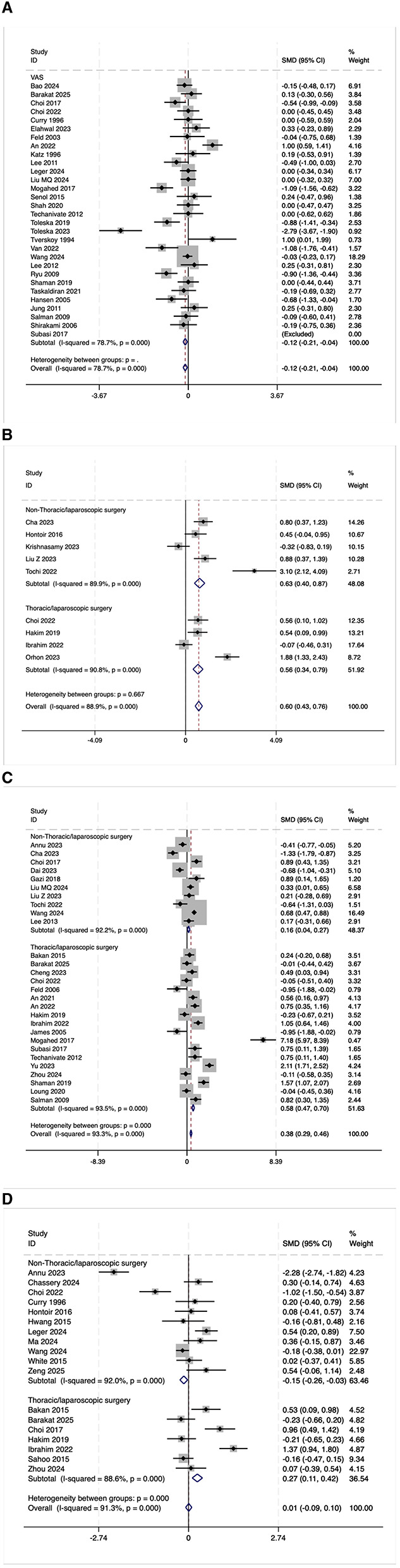
**(A)** The forest plot of the impact of OFA on VAS. **(B)** The forest plot of the impact of OFA on QOR-40. **(C)** The forest plot of the impact of OFA on postoperative extubation time. **(D)** The forest plot of the impact of OFA on postoperative PACU stay time.

The VAS scores 24 h after the operation were statistically analyzed in 30 trials. However, due to the high heterogeneity observed (*I*^2^ = 78.7%, Figure 10A), a meta-analysis was not conducted. Among these 30 studies, eight (27%) reported lower VAS scores in the OFA group, two (7%) reported higher scores, and 20 (66%) reported no significant difference between the groups. After excluding 12 low-quality studies (16, 21, 23–25, 27, 29–34), 18 high-quality studies were re-evaluated. In this subset, four (22%) reported lower VAS scores in the OFA group, one (6%) reported higher scores, and 13 (72%) reported no significant difference between the two groups. These findings suggest that although a subset of studies indicated a potential benefit of OFA in reducing postoperative pain, the majority of studies did not demonstrate a significant difference in VAS scores between the OFA and OBA groups.

#### 3.5.2 QoR-40

Nine randomized trials reported the overall score of QoR-40. Due to the high heterogeneity observed (*I*^2^ = 88.9%, Figure 10B), a meta-analysis was not conducted. Among the nine studies, seven (78%) reported higher QoR-40 scores in the OFA group, one (11%) reported lower scores, and one (11%) found no significant difference between the groups. After excluding two low-quality studies (15, 35), seven high-quality studies were re-evaluated. In this subset, six (86%) reported higher QoR-40 scores in the OFA group, zero (6%) reported lower scores, and one (14%) reported no significant difference between the two groups. These findings indicate that OFA may have certain advantages in terms of postoperative satisfaction.

#### 3.5.3 Postoperative extubation time

The postoperative extubation time was evaluated in 28 trials. Due to high heterogeneity (*I*^2^ = 93.3%, Figure 10C), a meta-analysis was not conducted. Among the 28 studies, five (18%) reported a shorter extubation time in the OFA group, 14 (50%) reported a longer time, and nine (32%) reported no significant difference between the two groups. After excluding five low-quality studies (15, 21, 27, 36, 37), 23 high-quality studies were re-evaluated. In this subset, three (13%) reported a shorter extubation time in the OFA group, 12 (52%) reported a longer time, and eight (35%) reported no significant difference between the two groups. Overall, half of the studies suggest that OFA may prolong the extubation time, and OFA was possibly associated with a longer extubation time.

#### 3.5.4 Postoperative PACU stay time

The postoperative PACU stay time was evaluated in 18 trials. Due to the high heterogeneity observed (*I*^2^ = 91.3%, Figure 10D), a meta-analysis was not conducted. Among the 18 studies, two (11%) reported a shorter PACU stay time in the OFA group, four (22%) reported a longer time, and 12 (67%) reported no significant difference between the two groups. After excluding seven low-quality studies (14, 18, 19, 21, 25, 27, 36), 11 high-quality studies were re-evaluated. In this subset, one (9%) reported a shorter PACU stay time in the OFA group, three (27%) reported a longer time, and seven (64%) reported no significant difference between the two groups. Most studies did not find a statistically significant difference in PACU stay time, and current evidence does not support a definitive advantage of either approach in this outcome.

## 4 Discussion

Enhanced recovery after surgery (ERAS) emphasizes reducing opioid use during hospitalization and minimizing opioid prescriptions after discharge to mitigate the side effects associated with their use (38). PONV is a common complication and is ranked among the five most undesirable surgical outcomes by patients. PONV not only compromises patient comfort but can also lead to serious complications such as dehydration, electrolyte imbalances, wound dehiscence, and aspiration, thereby increasing the medical burden. This meta-analysis demonstrates that compared with OBA, OFA significantly reduces the incidence of postoperative PONV and the need for rescue analgesia while improving QoR-40 scores. In the present study, we comprehensively assessed postoperative recovery quality using the QoR-40 score. The results showed a potential advantage of the OFA group in terms of postoperative satisfaction and overall recovery quality. Despite some heterogeneity, the majority of high-quality studies (86%) reported higher QoR-40 scores in the OFA group, indicating that patients experienced better comfort and functional status during the early postoperative recovery period. This finding underscores the potential of OFA to enhance patient-centered outcomes. Although there were no significant differences in length of hospital stay, VAS score, PACU stay duration, or intraoperative bradycardia, OFA might prolong extubation time; it exhibited potential benefits in reducing postoperative respiratory and intestinal dysfunction.

Previous studies have reported findings consistent with ours. For example, Zhang (39) demonstrated a significant reduction in postoperative PONV risk with OFA, supporting our results with a larger sample size and increased representativeness. Additionally, Zhang (40) found that OFA improved QoR-40 scores, which is consistent with and complemented by the more extensive data in our study. Alexander (41) reported that OFA significantly decreased intraoperative and postoperative adverse events, further strengthening the potential value of OFA in preventing and controlling postoperative adverse reactions. Meanwhile, Minke (5) combined VAS and NRS pain scores to reveal lower pain levels with OFA. Although these two scales are correlated, they have measured different aspects and thus cannot be used interchangeably. Finally, Cheng (42) found no significant difference in PACU stay time between OFA and OBA groups after laparoscopic surgery, which aligns with our findings.

Surgical factors that increase the risk of PONV primarily include surgical stimuli such as artificial pneumoperitoneum and traction reactions, as well as the use of anesthetic drugs (43, 44). Opioid drugs directly act on the μ-opioid receptors in the chemoreceptor trigger zone, thereby activating the vomiting reflex center and inducing vomiting (45). Studies have shown that the use of drugs such as dexmedetomidine and lidocaine during surgery, as well as the implementation of multimodal analgesic strategies, can effectively reduce the incidence of PONV (46). In laparoscopic surgery, the initial establishment of pneumoperitoneum can lead to rapid abdominal expansion, which in turn causes traction on mechanical receptors and increased synthesis of serotonin (5-HT), contributing to PONV (47). A retrospective analysis reported that laparoscopic surgery and prolonged operative time are independent predictors of high-risk PONV (48). Previous meta-analyses have predominantly focused on thoracoscopic (49) or laparoscopic (42) surgeries to evaluate the safety and efficacy of OFA, demonstrating that OFA may provide more significant analgesic and anti-PONV advantages in these minimally invasive procedures. This suggests that the type of surgery may be an important factor influencing the effectiveness of OFA. Both thoracoscopic and laparoscopic surgeries are minimally invasive endoscopic procedures. Minimally invasive endoscopic surgeries are characterized by less trauma, reduced intraoperative blood loss, shorter hospital stays, fewer severe postoperative complications, and faster recovery. These characteristics may influence postoperative recovery indicators under OFA, such as PONV, analgesic demand, and QoR-40 scores. Studies have shown that an operation time exceeding 1 h significantly increases the incidence of PONV (50). The average operation time for laparoscopic surgery is ~40 min longer than that for open surgery (51). For video-assisted thoracoscopic surgery (VATS), it is usually about 65.56 min longer than that for open surgery (52). Given that longer operation times are associated with higher PONV risk, treating these surgeries as a separate subgroup helps clarify whether OFA still holds an advantage in this context. Therefore, all studies were divided into two groups: the “thoracoscopic or laparoscopic group” and the “non-thoracoscopic or laparoscopic group” for analysis, to evaluate the effect of OFA accurately. Compared with previous meta-analyses, this study included a larger number of studies and, for the first time, conducted subgroup analyses based on whether the surgery was thoracoscopic or laparoscopic, providing preliminary evidence for the applicability of OFA in different surgical procedures. It should be noted that the current OFA protocol has not been standardized. Differences in the selection of anesthetic drugs (such as dexmedetomidine, lidocaine, etc.), dosage, and administration methods across studies may lead to significant fluctuations in outcome indicators, such as analgesic effect, risk of nausea and vomiting, and extubation time. Future research needs to develop a standardized plan for the use of anesthetic drugs to facilitate better implementation and promotion. The significant heterogeneity observed in some outcomes may not only be related to differences in study design and anesthesia regimens but also to the characteristics of the included patients and the complexity of the surgeries. In this study, subgroup and meta-regression analyses were conducted to explore the sources of heterogeneity in the effects of OFA vs. OBA on various postoperative outcomes. The results indicated that surgical type and anesthetic technique significantly influenced specific outcomes such as PONV and nausea. Specifically, thoracic and laparoscopic surgeries were associated with a higher risk of PONV, while intravenous inhalational anesthesia increased the risk of nausea and vomiting compared to TIVA. The use of nerve blocks did not show significant associations with any of the examined outcomes. These findings underscore the importance of considering surgical type and anesthetic technique when interpreting pooled results. They suggest that optimizing anesthesia and surgical management strategies could improve specific postoperative outcomes. Future research should focus on identifying additional factors contributing to the heterogeneity of other outcomes and on exploring interventions that can further enhance postoperative recovery. Furthermore, female patients are at a higher risk of developing PONV compared to male patients (53). Moreover, laparoscopic sleeve gastrectomy is often more complex than cholecystectomy. These differences in surgical complexity, combined with varying intraoperative and postoperative management strategies, can influence indicators such as analgesic needs, extubation time, and the speed of postoperative recovery, thereby leading to differences in postoperative outcome measures. Previous studies have directly pooled data for outcome indicators with high heterogeneity (*I*^2^ > 75%), which may compromise the reliability of the conclusions. In contrast, this study adopted a systematic review approach for such indicators, thereby avoiding the interference of heterogeneity on the combined results. This methodological approach not only enhances the scientific rigor of the conclusions but also strengthens the explanatory power and credibility of the research findings.

This study has several limitations that need to be acknowledged. First, the majority of current studies focus on short-term outcomes within 24 h post-surgery, leaving a gap in evidence regarding the long-term effects of OFA on outcomes such as quality of life and chronic pain. Second, although we analyzed the overall score of the QoR-40 scale, we did not conduct subgroup analyses of its five dimensions. This limitation restricts a more granular understanding of patient recovery quality. Third, despite being one of the most comprehensive meta-analyses on OFA to date, our search strategies may have missed some relevant studies. Finally, we did not conduct stratified analysis based on specific OFA and OBA protocols, which might have masked some clinically significant differences during the combined analysis. These indicators are crucial for assessing postoperative recovery and directly reflect patients' experiences and feelings.

In conclusion, OFA, as an anesthetic strategy for reducing opioid use, has demonstrated significant advantages in terms of postoperative comfort and recovery quality. Future studies should incorporate multi-dimensional subgroup analyses with larger sample sizes to enhance the persuasiveness and clinical relevance of the research conclusions. With the implementation of more high-quality research and the accumulation of practical experience, OFA is expected to be more widely applied in clinical anesthesia practice.

## Data Availability

The original contributions presented in the study are included in the article/Supplementary material, further inquiries can be directed to the corresponding authors.
