# Supplementary material for: Impact of the absence of opioid anesthesia on postoperative outcome indicators: a systematic review and meta-analysis
Source: Front Med (Lausanne). 2025 Aug 18;12:1639968. doi: 10.3389/fmed.2025.1639968 (PMC12399677; doi:10.3389/fmed.2025.1639968)
Supplement: Supplementary file 1 [file Table_1.docx]

**Supplementary Table 1** Search strategy

| Database | No. | Query | Result |
| --- | --- | --- | --- |
| Pubmed | #1 | (((Non opioid) OR ((((((((((((((((Analgesics, Non Narcotic[Title/Abstract]) OR (Non-Narcotic Analgesics[Title/Abstract])) OR (Non-Opioid Analgesic[Title/Abstract])) OR (Analgesic, Non-Opioid[Title/Abstract])) OR (Nonopioid Analgesic[Title/Abstract])) OR (Analgesic, Nonopioid[Title/Abstract])) OR (Non-Opioid Analgesics[Title/Abstract])) OR (Analgesics, Non-Opioid[Title/Abstract])) OR (Non Opioid Analgesics[Title/Abstract])) OR (Analgesics, Nonnarcotic[Title/Abstract])) OR (Nonnarcotic Analgesics[Title/Abstract])) OR (Analgesics, Nonopioid[Title/Abstract])) OR (Non Opioid Analgesic[Title/Abstract])) OR (Analgesic, Non Opioid[Title/Abstract])) OR (Opioid Analgesic, Non[Title/Abstract])) OR (Nonopioid Analgesics[Title/Abstract])) OR (opioid free[Title/Abstract]))) | 79913 |
|  | #2 | ((general anesthesia) OR (((Anesthesias, General[Title/Abstract]) OR (General Anesthesia[Title/Abstract])) OR (General Anesthesias[Title/Abstract])))) | 139252 |
|  | #3 | ((randomized controlled trial[Publication Type]) OR (randomized[Title/Abstract])) | 1052088 |
|  | #4 | #1 AND #2 AND #3 | 1459 |
| Cochrane | #1 | (Non opioid):ti,ab,kw | 6506 |
|  | #2 | (opioid free):ti,ab,kw | 1970 |
|  | #3 | (Analgesics, Non Narcotic):ti,ab,kw | 2919 |
|  | #4 | (Non-Narcotic Analgesics):ti,ab,kw | 2581 |
|  | #5 | (Non-Opioid Analgesic):ti,ab,kw | 853 |
|  | #6 | (Nonopioid Analgesic):ti,ab,kw | 1015 |
|  | #7 | (Analgesics, Nonnarcotic):ti,ab,kw | 2624 |
|  | #8 | (Opioid Analgesic, Non):ti,ab,kw | 4582 |
|  | #9 | #1 OR #2 OR #3 OR #4 OR #5 OR #6 OR #7 OR #8 | 10500 |
|  | #10 | (general anesthesia):ti,ab,kw | 39026 |
|  | #11 | (Anesthesias, General):ti,ab,kw | 39012 |
|  | #12 | (General Anesthesias):ti,ab,kw | 39012 |
|  | #13 | #10 OR #11 OR #12 | 39026 |
|  | #14 | (randomized controlled):ti,ab,kw | 1100391 |
|  | #15 | (randomized):ti,ab,kw | 1403484 |
|  | #16 | #14 OR #15 | 1403484 |
|  | #17 | #9 AND #13 AND #16 | 1071 |
| Web of science | #1 | TS=((Non opioid) OR (Analgesics, Non Narcotic) OR (Non-Narcotic Analgesics) OR (Non-Opioid Analgesic) OR (Analgesic, Non-Opioid) OR (Nonopioid Analgesic) OR (Analgesic, Nonopioid) OR (Non-Opioid Analgesics) OR (Analgesics, Non-Opioid) OR (Non Opioid Analgesics) OR (Analgesics, Nonnarcotic) OR (Nonnarcotic Analgesics) OR (Analgesics, Nonopioid) OR (Non Opioid Analgesic) OR (Analgesic, Non Opioid) OR (Opioid Analgesic, Non) OR (Nonopioid Analgesics) OR (opioid free)) | 63312 |
|  | #2 | TS=(general anesthesia OR Anesthesias, General OR General Anesthesia OR General Anesthesias) | 231184 |
|  | #3 | TS=(randomized controlled trial OR randomized) | 1647983 |
|  | #4 | #1 AND #2 AND #3 | 1121 |
| Embase | #1 | 'opioid-free anesthesia'/exp | 606 |
|  | #2 | 'analgesics, non narcotic':ab,ti | 3 |
|  | #3 | 'non-narcotic analgesics':ab,ti | 365 |
|  | 4 | 'non-opioid analgesic':ab,ti | 797 |
|  | 5 | 'analgesic, non-opioid':ab,ti | 4 |
|  | 6 | 'nonopioid analgesic':ab,ti | 380 |
|  | 7 | 'non-opioid analgesics':ab,ti | 1466 |
|  | 8 | 'analgesics, non-opioid':ab,ti | 25 |
|  | 9 | 'non opioid analgesics':ab,ti | 1445 |
|  | 10 | 'non opioid analgesic':ab,ti | 796 |
|  | 11 | 'nonopioid analgesics':ab,ti | 652 |
|  | 12 | #1 OR #2 OR #3 OR #4 OR #5 OR #6 OR #7 OR #8 OR #9 OR #10 OR #11 | 3972 |
|  | 13 | 'general anesthesia':ab,ti | 80799 |
|  | 14 | 'anesthesias, general':ab,ti | 2 |
|  | 15 | 'general anesthesia':ab,ti | 80799 |
|  | 16 | #13 OR #14 OR #15 | 80800 |
|  | 17 | 'randomized controlled trial':ab,ti | 201028 |
|  | 18 | 'randomized':ab,ti | 1284516 |
|  | 19 | #17 OR #18 | 1284516 |
|  | 20 | #12 AND #16 AND #19 | 95 |
