# Supplementary material for: Impact of the absence of opioid anesthesia on postoperative outcome indicators: a systematic review and meta-analysis
Source: Front Med (Lausanne). 2025 Aug 18;12:1639968. doi: 10.3389/fmed.2025.1639968 (PMC12399677; doi:10.3389/fmed.2025.1639968)
Supplement: Supplementary file 2 [file Table_2.docx]

**Supplementary Table 2** Detailed Anesthesia Protocols

| First author/year | Detailed intraoperative regimen in opioid-free anesthesia group | Detailed intraoperative regimen in control group |
| --- | --- | --- |
| Barakat [15] 2025 | Dexmedetomidine 0.5 mcg/kg and 1 mg/kg lidocaine over 10 min starting 10 min before induction. General anesthesia was intravenously induced using propofol (2 mg/kg), rocuronium (0.6 mg/kg), and ketamine (0.15 mg/kg). Anesthesia was maintained with sevofurane (dial setting regulated to achieve a minimum alveolar concentration between 1 and 1.2 for the remaining duration of the surgery according to the mean age of the patients who all fall in the same age category) and a mixture of medical air and oxygen, intravenous infusion of 0.3 mcg/kg/h dexmedetomidine, 1.5 mg/kg/h lidocaine and 0.15 mg/kg/h ketamine. | Propofol (2 mg/kg), fentanyl (2 mcg/kg), ketamine (0.15  mg/kg), and rocuronium (0.6 mg/kg). Remifentanil IV infusion was initiated with a dose range of 0.2–0.3 mcg/kg/min, depending on the patient’s hemodynamic status. Anesthesia was maintained with sevofurane/medical air/oxygen (as described above), remifentanil, and 0.15 mg/kg/h ketamine. Te dosage calculation of the used drugs was based on the adjusted body weight (ABW) |
| Zeng [16] 2025 | Due to the challenges of cooperation in the pediatric population, all children were induced with 8% sevoflurane combined with 4 L/ min oxygen inhalation anesthesia after entering the operating room. After the loss of consciousness, vital signs were monitored, and venous channels were established. Propofol (2-3 mg/kg) and cisatracurium (2 mg/kg) were injected intravenously for general anesthesia induction. Both groups of patients received propofol 6 to 9 mg/kg/h and 2% to 3% sevoflurane via inhalation to maintain anesthesia during surgery, a single dose of 0.5 mg/kg esketamine was administered for induction. The use of 0.5 mcg/kg/min dexmedetomidine pump injection was maintained for 10 minutes, after which the dose was changed to 0.2 mg/kg/h for maintenance anesthesia | Due to the challenges of cooperation in the pediatric population, all children were induced with 8% sevoflurane combined with 4 L/ min oxygen inhalation anesthesia after entering the operating room. After the loss of consciousness, vital signs were monitored, and venous channels were established. Propofol (2-3 mg/kg) and cisatracurium (2 mg/kg) were injected intravenously for general anesthesia induction. Both groups of patients received propofol 6 to 9 mg/kg/h and 2% to 3% sevoflurane via inhalation to maintain anesthesia during surgery, Fentanyl was added during induction at 2 mg/kg, and anesthesia was maintained with 0.2 to 0.3 mg/kg/min remifentanil. |
| Bao [17] 2024 | A loading dose of dexmedetomidine (0.7µg/kg over 10min), dexamethasone (5mg), midazolam (0.05mg/kg), propofol (2–3mg/kg), rocuronium (1mg/kg), lidocaine (1.5mg/kg) and magnesium sulfate (2.5g), based on previous studies.22–25 The maintenance medications included propofol (4–6mg/kg/hour), lidocaine (1.0mg/kg/hour), dexmedetomidine (0.5µg/kg/hour) and cisatracurium (0.05mg/kg/hour) | A conventional strategy with opioids for anaesthesia. The induction medications included a loading dose of dexmedetomidine (0.7µg/kg over 10min), dexamethasone (5mg), midazolam (0.05mg/kg), propofol (2–3mg/kg), rocuronium (1mg/kg) and sufentanil (0.5µg/kg). The maintenance medications included propofol (4–6mg/kg/hour), remifentanil (0.2µg/kg/min), dexmedetomidine (0.5µg/kg/hour) and cisatracurium (0.05mg/kg/hour) |
| Chassery [18] 2024 | A preoperative Dex infusion (1 mg/kg; i.v.) in 100 ml of normal saline over 30 min was administered and a normal saline injection (2 ml; i.v.) was performed on induction of anaesthesia and If requested during the surgery, at the discretion of the physician in charge, a rescue analgesic could be administered with Dex infusion (0.4 mg/kg; i.v.) in 100 ml of normal saline over 30 min and a normal saline injection (1 ml; i.v.) | A 100 ml preoperative normal saline infusion over 30 min was administered and a dose of sufentanil 10 mg, 2 ml i.v. was injected on induction of anaesthesia and If requested during the surgery, at the discretion of thephysician in charge, a rescue analgesic could be administered with a dose of sufentanil 5 mg, 1 ml i.v. and a normal saline infusion of 100 ml over 30 min |
| Copik [19] 2024 | PVB and induction to general anesthesia was completed,Bolus dose of lidocaine 1.5 mg/kg i.v. followed by a continuous infusion at a dose of 2.0 mg kg/h for 2 hours, then reduced to 1.2 mg kg/h and maintained  throughout the study period, meaning 24 hours postoperatively.Bolus dose of ketamine 0.35 mg/kg i.v. followed by a continuous infusion at a dose of 0.2 mg kg/h for 2 hours, then reduced to 0.12 mg kg/h  and maintained throughout the study period, meaning 24 hours postoperatively | PVB and induction to general anesthesia was completed, a dose of 1.5 µg/kg was used for anesthesia induction, and subsequently, fractional doses of fentanyl 1 to 3 µg/kg were administered if the patient’s HR or mean blood pressure (MBP) rose more than 20% above the baseline value obtained just before surgery commenced, which was predefined as a sign of experiencing pain. |
| Leger [20] 2024 | The OFA protocol was started before surgery with a clonidine infusion at an initial rate of 50 μg/h and then adapted according to hemodynamic stability with a maximal rate of 150 μg/h (100 μg/h for patients with body weight of less than 50kg). Anesthesia was induced and maintained with hypnotic drugs and neuromuscular blocking agents at the anesthesiologist’s discretion. The OFA protocol also included at induction a magnesium sulfate infusion (40mg/kg), a lidocaine infusion (1.5-mg/kg bolus dose in 10min followed by 1.5-mg/kg/h continuous infusion), and a ketamine infusion (0.5-mg/kg bolus dose followed by a 0.2-mg/kg/h continuous infusion). The ketamine infusion had to be stopped 30min before the end of the surgery, the clonidine was stopped at the surgery end, and the lidocaine infusion was stopped after 1h in the postanesthesia care unit (PACU) | The standard protocol followed the most recent French guidelines on the use of analgesic drugs during anesthesia.The standard anesthesia protocol combined hypnotic drugs, neuromuscular blocking agents, and opioids (sufentanil or remifentanil). A low-dose ketamine bolus (0.15mg/kg) was allowed at anesthesia induction, possibly followed by repeated boluses as in our standard practice |
| Liu MQ [21] 2024 | Anesthesia was induced with propofol, dezocine, and dexmedetomidine (DEX) and maintained with propofol, DEX, and an intercostal nerve block, along with spontaneous breathing throughout the operation | Received propofol, fentanyl, and cisatracurium for anesthesia induction, and maintenance was achieved with propofol and remifentanil, along with mechanical ventilation during the operation |
| Ma [22] 2024 | Lidocaine (1–1.5 mg/kg bolus), ketamine (5 mg/kg/min), and dexmedetomidine (0.5–1.0 µg/kg/h) were continuously infused from induction until approximately one hour before the anticipated end of surgery,Anesthesia maintenance for both groups was achieved with sevoflurane (0.7–1.3 MAC) | Included fentanyl (1 mg/kg) during induction and redosed every 45 minutes until the end of surgery, supplemented as necessary,Anesthesia maintenance for both groups was achieved with sevoflurane (0.7–1.3 MAC) |
| Sarma [23] 2024 | General anesthesia was induced with injection propofol (1–2 mg/kg) and cisatracurium (0.1 mg/kg). After adequate muscle relaxation, supraglottic device (SAD) of appropriate size was placed. Dexamethasone 8 mg was given after induction. Anesthesia was maintained with 50% air, oxygen, and sevofurane titrated to achieve MAC between 0.8 and 1, ESP block and dexmedetomidine infusion 0.5 µg/kg/h was initiated 10 min prior to induction and continued between 0.3 and 0.7 µg/kg/h based on intraoperative hemodynamics. Magnesium sulfate 40 mg/kg was also infused slowly over 10 min after induction | General anesthesia was induced with injection propofol (1–2 mg/kg) and cisatracurium (0.1 mg/kg). After adequate muscle relaxation, supraglottic device (SAD) of appropriate size was placed. Dexamethasone 8 mg was given after induction. Anesthesia was maintained with 50% air, oxygen, and sevofurane titrated to achieve MAC between 0.8 and 1,fentanyl 2 µg/kg intravenous (IV) was given 5 min before induction and was repeated with 1 µg/kg after every hour intraoperatively |
| Wang [24] 2024 | Propofol 1.5–2.0 mg/kg,received intravenous esketamine 0.3 mg/kg and intravenous lidocaine 1 mg/kg,received dexmedetomidine infusion (0.5 ug/kg followed by 0.2 ug/kg/h until skin closure), After tracheal intubation with cisatracurium 0.15–0.2 mg/kg, the patients’ lungs were ventilated and received esketamine 0.1 mg/kg boluses | Propofol 1.5–2.0 mg/kg,intravenous sufentanil 0.3ug/kg and normal saline volume matched to lidocaine, After tracheal intubation with cisatracurium 0.15–0.2 mg/kg, the patients’ lungs were ventilated and sufentanil 0.1 ug/kg boluses |
| Zhou [25] 2024 | Esketamine 0.5 mg/kg (I.V injection) was administered after a bolus of dexmedetomidine 0.5 ug/kg was administered 10 minutes and included midazolam (0.05mg/kg), propofol (1–2 mg/kg, total body weight), and rocuronium (0.6 mg/kg) before endotracheal intubation and TAP, administered a continuous intravenous infusion containing dexmedetomidine (0.2–0.3 μg/kg/h), esketamine (0.3 mg/kg/h), propofol (2 to 3 mg/kg/h), and sevoflurane (with a MAC: minimum alveolar concentration, of 0.8–1), as well as cisatracurium besilate (0.04–0.05mg/kg/h) given intravenously | Sufentanil (0.2–0.3 ug/kg) and included midazolam (0.05mg/kg), propofol (1–2 mg/kg, total body weight), and rocuronium (0.6 mg/kg) before endotracheal intubation, received repetitive doses of sufentanil (0.3 μg/kg) and propofol (2 to 3 mg/kg/h) as needed, determined by the anesthesiologist, and the usage of sevoflurane as well as cisatracurium besilate is same to the OFA group |
| Annu [26] 2023 | Anaesthesia was induced with IV propofol until loss of response to verbal command, after giving preservative-free 2% IV lignocaine 1.5 mg/kg to prevent propofol-induced pain and to reduce the stress response. Subsequently, IV succinylcholine 1.5 mg/kg was administered, followed by tracheal intubation with an appropriately sized endotracheal tube. Anaesthesia was maintained with IV propofol infusion 3–6 mg/kg/h and ventilation with nitrous oxide in an oxygen mixture (66:33) and neuromuscular blockade with IV atracurium and given non-opioid analgesia with IV dexmedetomidine (1 µg/kg loading dose, 10 min before induction of general anaesthesia and 10 min before induction as slow IV over 10 min and 0.5 µg/kg/h infusion thereafter) and IV lignocaine (1.5 mg/kg loading just before induction and 1.5 mg/kg/h infusion later) | Anaesthesia was induced with IV propofol until loss of response to verbal command, after giving preservative-free 2% IV lignocaine 1.5 mg/kg to prevent propofol-induced pain and to reduce the stress response. Subsequently, IV succinylcholine 1.5 mg/kg was administered, followed by tracheal intubation with an appropriately sized endotracheal tube. Anaesthesia was maintained with IV propofol infusion 3–6 mg/kg/h and ventilation with nitrous oxide in an oxygen mixture (66:33) and neuromuscular blockade with IV atracurium and IV morphine (0.15 mg/kg) before induction of general anaesthesia, and they were started on normal saline infusion. Surgical wound site infiltration with 20 ml of 0.25% bupivacaine was given at the end for postoperative analgesia |
| Cha [27] 2023 | Either 1.5 mg/kg lidocaine and general anesthesia was standardized with 2.0 mg/kg propofol (Fresenius Kabi Deutschland GmbH, Homburg, Germany) and 1 mg/kg scoline | 0.3 µg/kg sufentanil was injected intravenously over a 3 s period with activation of the pump and general anesthesia was standardized with 2.0 mg?kg propofol and 1 mg/kg scoline |
| Cheng [28] 2023 | Esketamine (0.3–0.5  mg/kg i.v.) and patients received dexmedetomidine (0.5 μg/kg i.v.) in a 10-min period before induction followed by a continuous infusion with 0.1–0.3 μg/kg/min until the end of surgery and ultrasound-guided bilateral transversus abdominis plane (TAP) | sufentanil (0.2–0.4  μg/kg i.v.) and the same amount of normal saline was administered in the same way. Anesthesia was induced with midazolam (0.05 mg/kg i.v.), propofol (2–2.5  mg/kg i.v.), cis-atracurium (1–1.5  mg/kg i.v.) in all patients and ultrasound-guided bilateral transversus abdominis plane (TAP) |
| Dai [29] 2023 | Unilateral/bilateral QLB surgery and Anesthesia was induced and maintained with TCI of propofol 3 to 3.5 μg/mL and intravenous rocuronium 0.6mg/kg | Unilateral/bilateral QLB surgery and with TCI of propofol 3 to 3.5 μg/mL and remifentanil 2 to 4ng/mL and intravenous rocuronium 0.6mg/kg |
| Elahwal [30] 2023 | Received 0.03 mg/kg midazolam prior and propofol (loading 1.5 to 2.5 mg/kg then 50–200 µg/kg/min maintenance) and atracurium (0.5 mg/kg atracurium then increments 0.1 to 0.2 mg/kg) and received dexmedetomidine (loading 1 µg/kg,continuous intraoperative infusion 0.3–0.5 µg/ kg/h), lidocaine (loading 1.5 mg/kg, infusion 2 mg/kg/ h) and ketamine infusion 0.1–0.3 mg/kg/h | Received 0.03 mg/kg midazolam prior and propofol (loading 1.5 to 2.5 mg/kg then 50–200 µg/kg/min maintenance) and atracurium (0.5 mg/kg atracurium then increments 0.1 to 0.2 mg/kg) and received fentanyl (loading 1 mic/kg then continuous intraoperative infusion 0.5 µg/kg/h) and two syringes with normal saline 0.9% |
| Krishnasamy [31] 2023 | Received dexmedetomidine 1 mg/kg and a bolus of ketamine 0.3 mg/kg and lidocaine 1.5 mg/kg, propofol 2 mg/kg and the trachea was intubated with an appropriate-sized tube using 0.1 mg/kg vecuronium as a muscle relaxant. Patients in both groups were given deep serratus anterior plane block (D-SAPB) using 0.25% ropivacaine 20 mL with 8 mg dexamethasone under ultrasound (USG) guidance | Received fentanyl 2 mg/kg,propofol 2 mg/kg and the trachea was intubated with an appropriate-sized tube using 0.1 mg/kg vecuronium as a muscle relaxant. Patients in both groups were given deep serratus anterior plane block (D-SAPB) using 0.25% ropivacaine 20 mL with 8 mg dexamethasone under ultrasound (USG) guidance |
| Liu Z [32] 2023 | Premedicated with dexmedetomidine 1 μg/kg over 10 min, and then induced with etomidate 0.3 mg/kg, s-ketamine 0.5 mg/kg, lidocaine 1.5 mg/kg and maintained with dexmedetomidine 0.5 μg/kg/h, s-ketamine 0.25 mg/kg/h, and propofol 3-4 mg/kg/h,Rocuronium bromide 0.6 mg/kg was used in  both groups for muscle relaxation | General anesthesia was induced using etomidate 0.3 mg/kg and remifentanil 1-2 μg/kg and maintained with propofol 4-6 mg/kg/h and remifentanil 0.05–0.2 ug/kg/min,Rocuronium bromide 0.6 mg/kg was used in both groups for muscle relaxation |
| Orhon [33] 2023 | Anesthesia was induced with propofol (2 mg/kg), ketamine (2 mg/kg), and rocuronium (0.6 mg/kg). This group did not take any opioids during the operation but instead received continuous dexmedetomidine infusion at an initial dose of 0.4 µg/kg/h, and the dose was adjusted to keep the BIS value between 40 and 60 and the ANI between 50 and 70 | Anesthesia was induced with propofol (2 mg/kg), remifentanil (1 µg/kg), and rocuronium (0.6 mg/kg). This group received continuous remifentanil infusion with an initial dose of 0.5 µg/kg/h, and the dose was adjusted to keep BIS <50 and ANI >50 throughout the operation |
| Toleska [34] 2023 | Received the following before the induction to general anesthesia: dexamethasone at 0.1 mg/kg and 1 gr of paracetamol,Induction to general anesthesia was with lidocaine at 1 mg/kg, propofol at 2mg/kg, ketamine at 0.5 mg/kg and rocuronium bromide at 0.6 mg/kg. After intubation, intravenous continuous infusion with lidocaine was at 2 mg/kg/h, ketamine 0.2 mg/kg/h and magnesium 15 mg/kg/h loaded on and intermittently 0.25 % bupivacaine 2-3 ml every 30-45 minutes given in the epidural catheter during surgery | Received the following for induction to anesthesia: lidocaine at 1 mg/kg, fentanyl 100 at µgr, propofol at 2mg/kg and rocuronium bromide at 0.6 mg/kg. They intermittently received 50-100 µgr fentanyl intravenously and 0.25 % bupivacaine 2-3 ml every 30-45 minutes, given in the epidural catheter during surgery |
| Yan [35] 2023 | 0.5–1 µg/kg intravenous dexmedetomidine was given before induction and propofol was set at 4 µg/mL, 0.9 mg/kg rocuronium and 0.125 mg/kg esketamine,total intravenous anesthesia was administered to all patients throughout the surgery. The target Cp of propofol was set at 3–4.0 µg/mL to maintain a BIS value between 45 and 60. An intermittent bolus of rocuronium was given to maintain muscle relaxation during the surgery and epidural administration | propofol was set at 3 µg/mL and 4 µg/kg fentanyl, Total intravenous anesthesia was administered to all patients throughout the surgery. The target Cp of propofol was set at 3–4.0 µg/mL to maintain a BIS value between 45 and 60. An intermittent bolus of ocuronium was given to maintain muscle relaxation during the surgery and epidural administration |
| Yu [36] 2023 | Received an infusion of dexmedetomidine 0.6 µg/kg at a constant rate for 10 min, propofol 2–3 mg/kg, lidocaine 1.5 mg/kg and cisatracurium besilate 0.2 mg/kg, infusion of 3– 12 mg/kg/h of propofol was administered and received a preincisional infiltration of 0.4 μg/kg dexmedetomidine mixed with 0.5% ropivacaine | Induced with a fixed protocol of 2–3 mg/kg propofol, 1 µg/kg remifentanil (followed by continuous intravenous infusion of 0.1–0.3 µg/kg/min), and 0.2 mg/kg cisatracurium besilate, infusion of 3–12 mg/kg/h of propofol was administered and received 1 μg/kg dexmedetomidine mixed with 0.5% ropivacaine |
| Choi [37] 2022 | A loading dose of IV dexmedetomidine 0.7 μg/kg for 10 min before the induction of general anesthesia, followed by a continuous infusion of 0.5 μg/kg/h, which was adjusted in steps of 0.1 μg/kg/h,Immediately after induction of anesthesia, a bolus dose of IV lidocaine 1.5 mg/kg was administered, followed by a continuous dose of 1.5 mg/kg/h. Dexmedetomidine and lidocaine infusions were stopped at the beginning of skin suturing | A continuous infusion of IV remifentanil targeting an effector site concentration of 3.5 ng/mL using a target-controlled infusion (Orchestra Base Primea, Fresenius Vial, Brezins, France) before the induction of general anesthesia. After induction of anesthesia, the remifentanil infusion was adjusted in increments of 0.5 ng/mL AND Remifentanil infusion was stopped at the end of the skin suturing. |
| An [38] 2022 | With dexmedetomidine (loading dose with 0.6 μg/kg for 10 min and then 0.5 μg/kg/h continuous infusion) and sevofurane plus bilateral paravertebral blockade (0.2 μg/kg dexmedetomidine and 0.5% ropivacaine 15 ml per side) | With remifentanil, sevofurane, and bilateral paravertebral blockade (0.5% ropivacaine 15 ml per side) |
| Ibrahim [39] 2022 | Premedicated with IV dexmedetomidine 0.1μg/kg in 100ml normal saline over 10min then induced with propofol (2mg/kg) ketamine (0.5mg/kg) mixture and maintained on dexmedetomidine 0.5μ./kg/h, ketamine 0.5mg/kg/h, and lidocaine 1mg/kg/h were prepared in 50ml normal saline to run at a rate of 50ml/h and Cisatracurium (0.15mg/kg) was used in both groups and OSTAP | General anaesthesia was induced using IV propofol 2mg/kg and fentanyl 1μg/kg and Cisatracurium (0.15mg/kg) was used in both groups and OSTAP |
| Menck [40] 2022 | Received magnesium sulfate (40 mg/kg), ketamine (25 mg), lidocaine (1.5−2 mg/kg), and dexmedetomidine (0.5 mcg/kg), general anesthesia was performed with propofol until loss of consciousness and rocuronium 1.2 mg/kg | Administered fentanyl 2.5 mcg.kg-1 in bolus,general anesthesia was performed with propofol until loss of consciousness and rocuronium 1.2mg/kg |
| Saravanaperumal [41] 2022 | Received 0.5 µg/kg of dexmedetomidine over 10 min in 100 ml of normal saline as infusion, about 10 min prior to procedure. At the start of the procedure, another 0.5 µg/kg of dexmedetomidine was given as infusion over 15 min and Propofol 1.5 mg/kg was given initially | Received 1 µg/kg of fentanyl over 10 min in 100 ml of normal saline as infusion, about 10 min prior to procedure. At the start of the procedure, another 1 µg/kg of fentanyl was given and Propofol 1.5 mg/kg was given initially |
| Tochie [42] 2022 | Entailed the intravenous (IV) magnesium sulfate, lidocaine, ketamine, dexamethasone, propofol, and rocuronium, followed by isofurane and a continuous infusion of a calibrated mixture of magnesium sulfate,  ketamine and clonidine | IV dexamethasone, diazepam, fentanyl, propofol, and rocuronium, followed by isofurane and reinjections of fentanyl propofol and a continuous infusion of normal saline as placebo |
| Toleska [43] 2022 | Received dexamethasone at 0.1 mg/kg and 1 g of paracetamol before introduction to anesthesia as a pre-emptive analgesia. Introduction to anesthesia consisted of giving midazolam at 0.04 mg/kg, lidocaine at  1 mg/kg, propofol at 2 mg/kg, ketamine at 0.5 mg/kg, and 0.6 mg/kg of rocuronium bromide. Immediately after intubation, continuous intravenous infusion with lidocaine at 2 mg/kg/h and magnesium sulfate at 1.5 g/h was given | A consisted of giving midazolam at 0.04 mg/kg, fentanyl at 0.002 mg/kg, 2 mg/kg of propofol and 0.6 mg/kg of rocuronium bromide. These patients received fractionated bolus doses of fentanyl during surgery. Prior to general anesthesia these patients did not receive dexamethasone |
| Van [44] 2022 | Received opioid-free anesthesia using dexmedetomidine in a loading dose of 0.25 mcg/kg before incision followed by a continuous infusion of 0.1 mg/kg/h during surgery; Lidocaine in a loading dose of 1 mg/kg before incision followed by a continuous infusion of 1 mg/kg/h during surgery; Esketamine in a loading dose of 25 mg before incision followed by a continuous infusion of 0.05 mg/kg/h during surgery. All the above adapted  up to a 50% increase or decrease at the discretion of the attending anesthesiologist. Furthermore, a loading dose of 2.5 gr Magnesium | Received opioid anesthesia using Sufentanil in a loading dose of 15- 25 mg Sufentanil before incision, followed by additional 5-10 mcg Sufentanil at the discretion of the attending anesthesiologist |
| Beloeil [45] 2021 | IV dexmedetomidine administered at the infusion rate of 0.4 to 1.4 μg/kg/h (dexmedetomidine group) and all patients received propofol (1.5 to 2mg/kg) and then desflurane, IV lidocaine (1.5mg/kg bolus plus1.5mg/kg/h), IV ketamine (0.5mg/kg bolus plus 0.25mg/kg/h), neuromuscular blockade, and dexamethasone (8mg, IV bolus) | Target-controlled infusion mode (3 to 5 ng/ml corresponding to 0.1 to 0.25 µg/kg/min; remifentanil group) and all patients received propofol (1.5 to 2mg/kg) and then desflurane, IV lidocaine (1.5mg/kg bolus plus 1.5mg/kg/h), IV ketamine (0.5mg/kg bolus plus 0.25mg/kg/h), neuromuscular blockade, and dexamethasone (8mg, IV bolus) |
| An [46] 2021 | With dexmedetomidine, sevoflurane plus thoracic paravertebral blockade and with a bolus of etomidate (0.2–0.3mg/kg), followed by cisatracurium 0.2mg/kg for muscle relaxation. After that, a single-lumen endotracheal tube (ETT) and a bronchial blocker (BB) were inserted into airway in turn. Muscle relaxation was  maintained with intermittent intravenous injection boluses of cisatracurium (2–4 mg per 30 min). Sevoflurane was adjusted to maintain anesthesia | With remifentanil, sevoflurane, and thoracic paravertebral blockade and with a bolus of etomidate (0.2–0.3 mg/kg), followed by cisatracurium 0.2mg/kg for muscle relaxation. After that, a single-lumen endotracheal tube (ETT) and a bronchial blocker (BB) were inserted into airway in turn. Muscle relaxation was maintained with intermittent intravenous injection boluses of cisatracurium (2–4 mg per 30 min). Sevoflurane was adjusted to maintain anesthesia |
| Taskaldiran [47] 2021 | Propofol (2-3 mg/kg), fentanyl (1 mg/kg), rocuronium (0.6 mg/kg), and lidocaine (1 mg/kg) were administered intravenously to both groups. For the maintenance of general anesthesia, sevoflurane (1-2%), oxygen (40%), and air (60%) were used and Erector Spinae Plane Block | Propofol (2-3 mg/kg), fentanyl (1 mg/kg), rocuronium (0.6 mg/kg), and lidocaine (1 mg/kg) were administered intravenously to both groups. For the maintenance of general anesthesia, sevoflurane (1-2%), oxygen (40%), and air (60%) were used,given intravenous remifentanil infusion (0.25-1.0 mcg/kg/min).For  postoperative analgesia, intravenous paracetamol (1 g) and tramadol (1 mg/kg) were administered 30 minutes before the termination of the operation. Prior to extubation, both groups were given sugammadex (2 mg/kg) |
| Shah [48] 2020 | Received dexmedetomidine loading infusion (1 µg/kg over 10 min) followed by anaesthetic induction with propofol, IVesmolol (0.5 mg/kg) to block the haemodynamic response to endotracheal intubation, facilitated with atracurium 0.6 mg/kg and pre-emptive IVparacetamol 1000 mg. Pre-incisional ultrasound-guided PECS-blocks preceded pre-incisional analgesic-dose IVketamine (0.5 mg/kg). BIS-guided dexmedetomidine infusion(no inhalational anaesthetics) maintained BIS in 40-60 range. If BIS approached 58-60, 2 ml dexmedetomidine (4 µg/ml) bolus, besides the baseline infusion of 0.6 µg/kg/h, was instituted. If after 2 min BIS did not fall, another 2 ml dexmedetomidine bolus, followed 2 min later by 20 mg propofol, if required, was administered | Received fentanyl 2 µg/kg, followed by propofol till loss of response to verbal command and atracurium 0.6 mg/kg to facilitate endotracheal intubation. Maintenance of anaesthesia comprised pre-emptive IVparacetamol 1000 mg, IVmorphine 0.1 mg/kg and sevoflurane (in medical-air; oxygen) to maintain  BIS in 40-60 range |
| Loung [49] 2020 | Administered lidocaine (2 mg/kg before induction and 1.5 mg/kg/h for maintenance), magnesium (30 mg/kg before induction and 1.5 g infusion for maintenance) combined with Intravenous (IV) injection of ketamine (0.5 mg/kg), and ketorolac (30 mg),Both groups received total intravenous anesthesia by propofol | Provided with IV fentanyl (5 mcg/kg for induction and 1.5 mcg/kg every 30 minutes for maintenance of anesthesia),Both groups received total intravenous anesthesia by propofol |
| Hakim [50] 2019 | Received IV dexmedetomidine 0.6 µg/kg loading over 5 min and anesthesia was induced with propofol 2 µg/kg and cisatracurium 0.1 mg/kg followed by endotracheal intubation and anesthesia was maintained with propofol infusion at 5–10 mg/kg/h,dexmedetomidine infusion at 0.2 µg/kg/h | Received IV fentanyl 1 µg/kg loading over 5 min and anesthesia was induced with propofol 2 µg/kg and cisatracurium 0.1 mg/kg followed by endotracheal intubation and anesthesia was maintained with propofol infusion at 5–10 mg/kg/h,fentanyl infusion at 0.5 µg/kg/h |
| Toleska [51] 2019 | Received dexasone (dexamethasone) 0.1 mg/kg and 1 g paracetamol intravenously (i.v.) as a preemptive analgesia. The induction to general endotracheal anesthesia was followed by administration of midazolam 0.04 mg/kg, lidocaine 1 mg/kg, propofol 2 mg/kg and rocuronium bromide 0.6 mg/kg aAnd ketamine 0.5 mg/kg  was given and intravenous continuous infusion with lidocaine 2 mg/kg/h and magnesium sulphate 1.5 g/hr was started | Introduced into general endotracheal anesthesia with midazolam 0.04 mg/kg, opioid (fentanyl) 0.002 mg/kg, propofol 2 mg/kg and rocuronium bromide 0.6 mg/kg,During the surgery, patients from Fentanyl group received fractionated bolus doses of fentanyl |
| Shaman [52] 2019 | After a loading dose of dexmedetomidine 0.5 µg/kg over 10 min, anesthesia was induced with propofol 2.5 − 3.5mg/kg, followed by atracurium 0.5 mg/kg, and maintained with continuous infusion of propofol 50 − 200 µg/kg/min and dexmedetomidine 0.1 − 0.3 µg/kg/h for maintaining BIS between 40 − 60. Lignocaine 1.5 mg/kg was administered at induction and an infusion of 0.1mg/kg/h was started immediately after the loading dose. Ketamine 0.5 mg/kg was given before incision | Anesthesia was induced with fentanyl 2 µg/kg and propofol 2.5 − 3.5 mg/kg, followed by atracurium 0.5 mg/kg for tracheal intubation, and maintained with continuous infusion of propofol 50 − 200 µg/kg/min and intermittent fentanyl 0.5 µg/kg bolus for maintaining bispectral index (BIS) between 40 and 60 |
| Gazi [53] 2018 | Received 1 μg/kg of dexmedetomidine and 2 mg/kg of propofol and 0.5 mg/kg of rocuronium were administered for induction of anesthesia | Received 1 μg/kg of remifentanil and 2 mg/kg of propofol and 0.5 mg/kg of rocuronium were administered for induction of anesthesia |
| Choi [54] 2017 | The dexmedetomidine group, who received an initial loading dose of dexmedetomidine (1 μg/kg over 10 min) during the induction of anesthesia, followed by a continuous infusion at a rate of 0.3–0.5 μg/kg/h | the remifentanil group , who received remifentanil at an initial target effect site concentration of 4 ng/ml during the induction of anesthesia, followed by a target effect site concentration of 2–3 ng/ml |
| Mogahed [55] 2017 | General anesthesia induction with propofol 2.5 mg/kg rocuronium bromide 0.6 mgkg and sevoflurane 1-1.5 MAC,received dexmedetomidine (Precedex, 200 ug per 2 mL; Abbott, USA) (an initial loading dose of 0.7 ug/kg given for a 10 min period followed by 0.4 ug/kg/h) | General anesthesia induction with propofol 2.5 mg/kg rocuronium bromide 0.6 mgkg and sevoflurane 1-1.5 MAC,received an intravenous (i.v.) remifentanil (an initial loading dose of 0.7 ug/kg given for a 10 min period followed by 0.2 ug/kg/h) |
| Subasi [56] 2017 | Endotracheal intubation was performed after inducing anesthesia with 2.5 mg/kg propofol, 0.6 mg/kg rocuronium (Esmeron, Organon, Netherlands), 1μg/ kg fentanyl (Fentanylcitrate, Abbott, USA). During maintenance, 150 μg/kg/min propofol and 0.5 µg/kg/h dexmedetomidine infusions were pumped using two different pumps at each cannulation sites. After the 5th minute of infusion, dexmedetomidine infusion rate was lowered to 0.3 µg/kg/h | Endotracheal intubation was performed after inducing anesthesia using 2.5 mg/kg propofol, 0.6 mg/kg rocuronium, 1μg/kg fentanyl. During maintenance, 150 μg/kg/min propofol and 0.5 µg/kg/min remifentanil infusions were pumped using two different pumps at two different venous cannulation site. After the  5th minute of infusion, remifentanil infusion was lowered to 0.3 µg/kg/min |
| Hontoir [57] 2016 | Received a loading dose of clonidine (0.2 µg/kg) and received a bolus of ketamine (0.3 mg/kg), lidocaine (1.5 mg/kg), and propofol (2-3 mg/kg) | Target-controlled infusion of rémifentanil and received a bolus of ketamine (0.3 mg/kg), lidocaine (1.5 mg/kg), and propofol (2-3 mg/kg) |
| Choi [58] 2016 | Induction was accomplished with full preoxygenation, 1% lidocaine (40 mg) and 1% propofol (2 mg/kg), followed by rocuronium (0.6 mg/kg). Anesthesia was maintained with O2 at 2 L/min, N2O at 3 L/min, 6-7 vol% of desflurane,and rocuronium was given if required and a loading dose of dexmedetomidine (1 µg/kg) was given over 10 minutes followed by a continuous infusion of 0.5 µg/kg/hr at the end of main procedures of the operation to the time in the PACU | Induction was accomplished with full preoxygenation, 1% lidocaine (40 mg) and 1% propofol (2 mg/kg), followed by rocuronium (0.6 mg/kg). Anesthesia was maintained with O2 at 2 L/min, N2O at 3 L/min, 6-7 vol% of desflurane,and rocuronium was given if required and given a loading dose of remifentanil (1.0 µg/kg) over 1 minute followed by continuous infusion of 0.08 µg/kg/min |
| Bakan [59] 2015 | Received 0.6ug/kg dexmedetomidine (loading-1) diluted to a total volume of 10 mL and infused in 10 min and dexmedetomidine in 1ug/mL, Lidocaine at 1.5 mg/kg, Lidocaine (20 mg/mL) or normal saline infusions (infusion-2) at 0.1 mL/kg/h and propofol infusion at 10 mg/kg/h was started immediately after loading doses. Vecuroniumat 0.1 mg/kg IV was given to facilitate tracheal intubation | Received fentanyl 2ug/kg in the same fashion and remifentanil in 50 ug/mL and propofol at 1.5 mg/kg was administered, Lidocaine (20 mg/mL) or normal saline infusions (infusion-2) at 0.1 mL/kg/h and propofol infusion at 10 mg/kg/h was started immediately after loading doses. Vecuroniumat 0.1 mg/kg IV was given to facilitate tracheal intubation |
| Hwang [60] 2015 | 0.01 μg/kg/min of dexmedetomidine (i.e., 0.5 μg/min for 50 kg patient) was administered continuously using a syringe pump, after 10 minutes of study drug infusion, 1–2 mg/kg of propofol,When the patients were fully sedated, 1 mg/kg of rocuronium was administered and the trachea was intubated after manual ventilation for 1 minute. Anesthesia was maintained using propofol at 3–12 mg/kg/h (i.e., 150–600 mg/hr for 50 kg patient) using TCI with dexmedetomidine at 0.01–0.02 μg/kg/min | 0.01 μg/ kg/min of remifentanil (i.e., 0.5 μg/min for 50 kg patient) was administered continuously using target-controlled infusion, after 10 minutes of study drug infusion, 1–2 mg/kg of propofol, When the patients were fully sedated, 1 mg/kg of rocuronium was administered and the trachea was intubated after manual ventilation for 1 minute. Anesthesia was maintained using propofol at 3–12 mg/kg/h (i.e.,150–600 mg/hr for 50 kg patient) using TCI with remifentanil at 0.01-0.2 μg/kg/min |
| Senol [61] 2015 | Received 0.015 mg/kg atropine sulphate and 0.07 mg/kg midazolam IM as a premedication 45 min before surgery. Anesthesia was induced with thiopental sodium 5 mg/kg and vecuronium 0.1 mg/kg iv and was maintained with 1 MAC desflurane and 70% nitrous oxide in oxygen. All patients received 1 g of intravenous paracetamol intraoperatively and administered normal saline 5 cc bolus and 10 cc/h infusion for induction and maintenance respectively | Received 0.015 mg/kg atropine sulphate and 0.07 mg/kg midazolam IM as a premedication 45 min before surgery. Anesthesia was induced with thiopental sodium 5 mg/kg and vecuronium 0.1 mg/kg iv and was maintained with 1 MAC desflurane and 70% nitrous oxide in oxygen. All patients received 1 g of intravenous paracetamol intraoperatively and administered remifentanil 1µg/kg iv bolus for induction and 0.25 µg/kg/min infusion for maintenance |
| White [62] 2015 | Administration of 2 ml of saline and anesthesia was induced with lidocaine 30–50 mg and propofol 2 mg/kg IV followed by the insertion of an LMA device. General anesthesia was maintained using a propofol infusion, 75 μg/kg/min, and desflurane (2–5% end-tidal) in 100% oxygen | Administration of 2 ml of fentanyl (100 μg) and anesthesia was induced with lidocaine 30–50 mg and propofol 2 mg/kg IV followed by the insertion of an LMA device. General anesthesia was maintained using a propofol infusion, 75 μg/kg/min, and desflurane (2–5% end-tidal) in 100% oxygen |
| Sahoo [63] 2015 | TCI whereas 0.01 µg/kg/min of Dexmedetomidine was administered continuously using a syringe pump in Dexmedetomidine group,Propofol was manually administered at the rate of 1 mg/kg followed immediately by an infusion of 10 mg/kg/hr. for 10 min, 8 mg/kg/hr. for the next 10 min and thereafter 6 mg/kg/hr. After 10 min. of administration of the study drug, when the patients were fully sedated 1 mg/kg of vercuronium was administered and the trachea was intubated with an approximately sized endotracheal tube after manual ventilation for 1 minute. Anaesthesia was maintained using propofol at 3–12 mg/kg/h and Dexmedetomidine at 0.05 µg/kg/min | Before induction ofanaesthesia Remifentanyl was administered at a dosage of 0.01 µg/kg/min continuously using target-controlled infusion,Propofol was manually administered at the rate of 1 mg/kg followed immediately by an infusion of 10 mg/kg/hr. for 10 min, 8 mg/kg/hr. for the next 10 min and thereafter 6 mg/kg/hr. After 10 min. of administration of the study drug, when the patients were fully sedated 1 mg/kg of vercuronium was administered and the trachea was intubated with an approximately sized endotracheal tube after manual ventilation for 1 minute. Anaesthesia was maintained using propofol at 3–12 mg/kg/h andremifentanyl at 0.05µg/kg/min |
| Mansour [64] 2013 | Co-induction of Propofol 2 mg/Kg and analgesic dose of Ketamine 0.5 mg/Kg, Rocuronium 0.5 mg/Kg followed by intubation,sevoflurane, 2‑4%, Ketamine infusion of 0.5 mg/Kg/h of ideal body weight | Co-induction of Propofol 2 mg/Kg and analgesic dose of Fentanyl 2-5 mcg/Kg, Rocuronium 0.5 mg/Kg followed by intubation,anesthesia was maintained by sevoflurane,2-4%, Fentanyl infusion of 0.025-0.25 mcg/Kg/min of ideal body weight |
| Lee [65] 2013 | Propofol 2-2.5mg/kg,was administered to induce anesthesia,which was maintained with desflurane.and received dexmedetomidine 1 ug/kg ,followed by 0.4-0.8 ug/kg per hour infusion during maintenance | Propofol 2-2.5mg/kg,was administered to induce anesthesia,which was maintained with desflurane.and received remifentanil 1ug/kg ,followed by 0.2-0.4 ug/kg per minute infusion during maintenance |
| Techanivate [66] 2012 | Anesthesia was induced with propofol (2 mg/Kg), and atracurium (0.5 mg/Kg) was given to facilitate laryngoscopy and endotracheal intubation. Anesthesia was maintained with desflurane in 60% nitrous oxide and 40% oxygen. After tracheal intubation, the present study drug (dexmedetomidine) was given by intravenously infusion for a period of 10 min with a syringe pump | Anesthesia was induced with propofol (2 mg/Kg), and atracurium (0.5 mg/Kg) was given to facilitate laryngoscopy and endotracheal intubation. Anesthesia was maintained with desflurane in 60% nitrous oxide and 40% oxygen. After trachealintubation, the present study drug (fentanyl) was given by intravenously infusion for a period of 10 min with a syringe pump |
| Lee [67] 2012 | With 0.2 mg glycopyrrolate i.m. and 2 mg midazolam i.m. 30 minutes before anesthesia induction,with 5 mg/kg thiopental sodium i.v,After loss of consciousness, 0.9 mg/kg rocuronium i.v. was given, and mask ventilation with 100% oxygen 8 L/min and 6 vol% desflurane was maintained for 3 minutes and maintained with 4-6 vol% desflurane, 1.5 L/min oxygen, and 1.5 L/min nitrous oxid | With 0.2 mg glycopyrrolate i.m. and 2 mg midazolam i.m. 30 minutes before anesthesia induction,with 5 mg/kg thiopental sodium i.v,After loss of consciousness, 0.9 mg/kg rocuronium i.v. was given, and mask ventilation with 100% oxygen 8 L/min and 6 vol% desflurane was maintained for 3 minutes and maintained with 4-6 vol% desflurane, 1.5 L/min oxygen, and 1.5 L/min nitrous oxid received 0.3 μg/kg/hr sufentanil infusion |
| Lee [68] 2011 | End-tidal sovoflurane concentration was maintained at 1 minimum alveolar concentration (MAC).The patients in sevoflurane group received an equal volume and of saline in place of remifentanil, the concentration of  sevoflurane was titrated to maintain a bispectral index in the range 40 to 60 | End-tidal sovoflurane concentration was maintained at 1 minimum alveolar concentration (MAC). Remifentanil was started at a rate of 0.1 μg/kg/min and subsequently stepwise by 0.05 μg/kg/min increments if inadequate analgesia was suspected |
| Jung [69] 2011 | Induction anaesthetic was accomplished with full preoxygenation, 1% lidocaine (40 mg) and 1% propofol (2 mg/kg), followed by rocuronium (0.6 mg/kg) all given intravenously (i.v.).Anaesthesia was maintained with oxygen at 2 l/min, nitrous oxide at 3 l/min and 6–7 vol.% of desflurane with rocuronium.received either a loading dose of dexmedetomidine 1 µg/kg i.v. administered over 10 min followed by a continuous infusion of 0.2 – 0.7 µg/kg per h | Induction anaesthetic was accomplished with full preoxygenation,1% lidocaine (40 mg) and 1% propofol (2 mg/kg), followed by rocuronium (0.6 mg/kg) all given intravenously (i.v.).Anaesthesia was maintained with oxygen at 2 l/min, nitrous oxide at 3 l/min and 6–7 vol.% of desflurane with rocuronium.a loading dose of remifentanil 0.8 – 1.2 µg/kg i.v. given over 1 min followed by continuous infusion of 0.05 – 0.1 µg/kg per min |
| De [70] 2010 | Received premedication with rectal midazolam 0.3 mg/kg and General anaesthesia was induced with a facemask, with increasing concentrations of sevoflurane in a mix of O2/N2O (33%/66%) to a final concentration of 4% and the intraoperative analgesia was performed bywrist blocks | The intraoperative analgesia was performed by titration with intravenous alfentanil in order to maintain blood pressure and cardiac frequency at less than 120% from baseline and to maintain spontaneous breathing. The postoperative analgesia began after the induction of anaesthesia with an injection of  intravenous propacetamol (30 mg/kg) and with rectal niflumic acid |
| Ryu [71] 2009 | Propofol 2 mg/kg was administered to induce anaesthesia, which was maintained using sevoflurane and received an i.v. magnesium sulphate bolus of 50 mg/kg followed by a 15 mg/kg/h continuous infusion | Propofol 2 mg/kg was administered to induce anaesthesia, which was maintained using sevoflurane and remifentanil titrated between 3 and 4 ng/ml using target-controlled infusion |
| Salman [72] 2009 | Anesthesia was induced with 2 mg/kg propofol. Vecuronium bromide (0.1 mg/kg) was used to facilitate tracheal intubation,received 1 µg/kg IV dexmedetomidine bolus dose in 10 minutes, and an infusion of 0.4 µg/kg/hr | Anesthesia was induced with 2 mg/kg propofol. Vecuronium bromide (0.1 mg/kg) was used to facilitate tracheal intubation,received 1 µg/kg IV remifentanil in 10 minutes, and an infusion of 0.2 µg/kg/min |
| Collard [73] 2007 | The preoperative medication was midazolam administered intravenously at a dose of 0.03mg/kg and After administration of remifentanil at a dose of 1ug/kg or fentanyl at a dose of 1ug/kg, propofol at a dose of 2.5mg/kg was induced by general anesthesia, and rocuronium at a dose of 0.8mg/kg was used for tracheal intubation | The preoperative medication was midazolam administered intravenously at a dose of 0.03mg/kg and After administration of esmolol at a dose of 1mg/kg, propofol at a dose of 2.5mg/kg was induced by general anesthesia, and rocuronium at a dose of 0.8mg/kg was used for tracheal intubation |
| Feld [74] 2006 | Dexmedetomidine (0.5 ug/kg) was given intravenously over 10 minutes to facilitate sedation with midazolam. A dose of up to 1 ug/kg dexmedetomidine,anesthesia was induced with lidocaine (100 mg),thiopental (1-4 mg/kg), and succinylcholine (0.6 mg/kg); dexmedetomidine was infused at a rate of 0.4 ug/kg/h | Anesthesia was induced with fentanyl (0.5 ug/kg), lidocaine (100 mg), thiopental (1-4 mg/kg), and succinylcholine (0.6 mg/kg). Anesthesia was maintained with fentanyl infusion (0.5 ug/kg/h) |
| Shirakami [75] 2006 | Anesthesia was induced with propofol 2mg/kg IV, and a laryngeal mask airway (LMA) was placed. Lidocaine 1mg/kg was administered IV just before the propofol IV injection to decrease injection pain. All patients received a suppository (diclofenac sodium, 50mg × 2; total, 100 mg) and local infiltration anesthesia (0.5% lidocaine, 200mg × 2; total, 400 mg) into the surgical field for pain relief before the start and the end of the operation and received the same volume of  saline IV during anesthesia | Anesthesia was induced with propofol 2mg/kg IV, and a laryngeal mask airway (LMA) was placed. Lidocaine 1mg/kg was administered IV just before the propofol IV injection to decrease injection pain. All patients received a suppository (diclofenac sodium, 50mg × 2; total, 100 mg) and local infiltration anesthesia (0.5% lidocaine, 200mg × 2; total, 400 mg) into the surgical field for pain relief before the start and the end of the operation and received fentanyl IV (25µg × 4; total,0.1mg; at anesthesia induction, before skin incision,before axillary dissection, and before skin closure) |
| James [76] 2005 | Given intravenously over 10 minutes to facilitate sedation with midazolam. A dose of up to 1 lg/kg dexmedetomidine.anesthesia was induced with lidocaine (100 mg),thiopental (1-4 mg/kg), and succinylcholine (0.6 mg/kg); dexmedetomidine was infused at a rate of 0.4 ug/kg/h | Anesthesia was induced with fentanyl (0.5 lg/kg), lidocaine(100 mg), thiopental (1-4 mg/kg), and succinylcholine(0.6 mg/kg). Anesthesia was maintained with fentanyl infusion (0.5 ug/kg/h), consistent with previous dosing. End-tidal desflurane concentration was adjusted to maintain the BIS at  45 to 50 during surgery |
| Hansen [77] 2005 | All patients received pre-medication with triazolam 0.125 mg and standard thoracic epidural anaesthesia and General anaesthesia was induced with thiopental 5 mg/kg and rocuronium 0.5 mg/kg and a tracheal tube was inserted. Anaesthesia was maintained with sevoflurane to a desired level of 0.8-1.0 minimal alveolar concentration (MAC) and placebo (sodium chloride 0.9%) | All patients received pre-medication with triazolam 0.125 mg and standard thoracic epidural anaesthesia and General anaesthesia was induced with thiopental 5 mg/kg and rocuronium 0.5 mg/kg and a tracheal tube was inserted. Anaesthesia was maintained with sevoflurane to a desired level of 0.8-1.0 minimal alveolar concentration (MAC) and remifentanil 0.4 mg/kg/min |
| Feld [78] 2003 | Ventilated with sevoflurane and anesthesia was supplemented with ketorolac, 30 mg iv given at the beginning and end of the case, clonidine, 300–500 µg iv starting within the first hour of anesthesia as tolerated for maintenance of blood pressure, lidocaine, 100 mg bolus given during induction of anesthesia followed by 4 mg/min for the first hour, 3 mg/min for the second hour and 2 mg/min until the end of the caseketamine, 0.17 mg/kg/h with a maximum dose of 1 mg/kg given with the iv saline fluid maintenance infusion, magnesium sulfate, 80 mg/kg total dose given in the iv saline fluid maintenance infusion during the case, and methylprednisolone, 60 mg given as an iv bolus in the holding area before the start of surgery | Received sevoflurane anesthesia supplemented with intermittent boluses of 50 µg fentanyl up to a total dose not to exceed 6 µg/kg of ideal body weight. Ideal body weight was calculated in men as: ideal body weight (in kilograms) = 50 ± 2.3 kg per 2.5 cm over 160 cm, women: ideal body weight (in kilograms) = 45.5 ± 2.3 kg per 2.5 cm over 160 cm |
| Curry [79] 1996 | Each patient was induced with propofol (starting with 2 mg/kg up to 3 mg/kg in 0.1% lidoCaine as needed). Vecuronium 0.1 mg/kg was used to facilitate intubation. General anesthesia was maintained with a propofol infusion (initiated at 200 pg/kg/min and titrated up to 300 pg/kg/min as needed), 60% nitrous oxide, and supplemental isoflurane not exceeding 0.5%. Muscle relaxation was reversed with neostigmine 0.07 mg/ kg and glycopyrrolate 0.2 m/kg and eceive the same saline | Each patient was induced with propofol (starting with 2 mg/kg up to 3 mg/kg in 0.1% lidoCaine as needed). Vecuronium 0.1 mg/kg was used to facilitate intubation. General anesthesia was maintained with a propofol infusion (initiated at 200 pg/kg/min and titrated up to 300 pg/kg/min as needed), 60% nitrous oxide, and supplemental isoflurane not exceeding 0.5%. Muscle relaxation was reversed with neostigmine 0.07 mg/ kg and glycopyrrolate 0.2 m/kg and eceive either fentanyl 1 pg/kg |
| Katz [80] 1996 | Midazolam and thiopentone and was maintained with isoflurane and 70%N20 in 02 (anesthesia wm induced with iv. midazolam (0.05 mg/kg) and thiopentone (3–5 mg/kg). Muscle relaxation and tracheal incubation were facilitated with vecuronium (0.1-0.15 mg/kg). Anesthesia was maintained with isoflurane and 70% N20 in 02. Opioids were not administered at induction or intra-operatively) | Midazolam and iv. aIfentanil (100,ug/kg), and was maintained with 70% N20 in 02, and an infusion of iv. alfentanil (1-2,ug/kg/min)(amsesthesia was induced with iv. midazolam(0.05 mg/kg) and iv. alfentanil (100pg/kg). Muscle relaxation and tracheal incubation were facilitated with vecuronium (0.1-0.15 mg/kg).Anesthesia was maintained with 70V0N O in 02 and a continuous infusion of iv. alfentanil (1–2ug/kg/min). The alfentanil dose was adjusted to maintain hemodynamic variables within 20% of pre-operative ward values by administering a bolus dose of iv. alfentanil (10-20pg/kg) and increasing the alfentanil infusion by increments of 0.25-O.5,ug/kg/min). The alfentanil infusion wa.. discontinued 30 min before. the anticipated end of surgery) |
| Sukhani [81] 1996 | Induction of anesthesia consisted of lidocaine 1 mg/kg IV, followed by propofol up to 2.5 mg/kg IV administered over l-2 min until loss of the eyelid reflex. The maintenance infusion of propofol was started at a rate of 200 pg/kg mini using a syringe-type infusion pump and Endotracheal intubation was facilitated  with atracurium 0.5 mg/kg IV, and anesthesia was maintained with a continuous infusion of propofol and nitrous oxide 66% in oxygen.similarly received 2 mL (60 mg) of ketorolac IV at the time of induction | Induction of anesthesia consisted of lidocaine 1 mg/kg IV, followed by propofol up to 2.5 mg/kg IV administered over l-2 min until loss of the eyelid reflex. The maintenance infusion of propofol was started at a rate of 200 pg/kg mini using a syringe-type infusion pump and Endotracheal intubation was facilitated with atracurium 0.5 mg/kg IV, and anesthesia was maintained with a continuous infusion of propofol and nitrous oxide 66% in oxygen.received 2 mL (100 pg) of fentanyl IV at the time of induction; |
| Tverskoy [82] 1994 | Anesthesia was induced with ketamine 2 mg/kg in combination with thiopental 3 mg/kg and maintained with isoflurane and ketamine 20 pg/kg/min | Anesthesia was induced with fentanyl 5 ug/kg combined with thiopental 3 mg/kg and maintained with isoflurane and fentanyl 0.02 ug/kg/min |

**OFA**: Opioid-Free Anesthesia; **OBA**: Opioid-Based Anesthesia; **PONV**: Postoperative Nausea and Vomiting; **LOS**: length of stay in hospital; **VAS**: Visual Analogue Scale; **NRS**: Numeric Rating Scale; **QoR-40**: Quality of Recovery-40; **OSTAP**: Oblique Subcostal Transversus Abdominis Plane block; **QLB**: Quadratus Lumborum Block; **TAP**: Transversus Abdominis Plane block; **D-SAPB**: Deep Serratus Anterior Plane Block.
