# Supplementary material for: Impact of the absence of opioid anesthesia on postoperative outcome indicators: a systematic review and meta-analysis
Source: Front Med (Lausanne). 2025 Aug 18;12:1639968. doi: 10.3389/fmed.2025.1639968 (PMC12399677; doi:10.3389/fmed.2025.1639968)
Supplement: Supplementary file 3 [file Table_3.docx]

**Supplementary Table 3** Risk of Bias Assessment of Included Studies Using the RoB 2 Tool

| First Author (Year) | Randomization process | Deviations from intended interventions | Missing outcome data | Measurement of the outcome | Selection of the reported result | Overall Bias |
| --- | --- | --- | --- | --- | --- | --- |
| Barakat [15] 2025 | Low risk | Some concerns | Low risk | Low risk | High risk | High risk |
| Zeng [16] 2025 | Low risk | Low risk | Low risk | Low risk | Low risk | Low risk |
| Bao [17] 2024 | Low risk | Low risk | Low risk | Low risk | Low risk | Low risk |
| Chassery [18] 2024 | Low risk | Low risk | Low risk | Some concerns | Low risk | Some concerns |
| Copik [19] 2024 | Low risk | Low risk | Low risk | Low risk | Low risk | Low risk |
| Leger [20] 2024 | Low risk | Low risk | Low risk | Low risk | Low risk | Low risk |
| Liu MQ [21] 2024 | Low risk | Low risk | Low risk | Low risk | Low risk | Low risk |
| Ma [22] 2024 | Low risk | Low risk | Low risk | Low risk | Low risk | Low risk |
| Sarma [23] 2024 | Low risk | Low risk | Low risk | Low risk | Low risk | Low risk |
| Wang [24] 2024 | Low risk | Low risk | Low risk | Low risk | Low risk | Low risk |
| Zhou [25] 2024 | Low risk | Low risk | Low risk | Low risk | Low risk | Low risk |
| Annu [26] 2023 | Low risk | Low risk | Low risk | High risk | Low risk | High risk |
| Cha [27] 2023 | Some concerns | Low risk | Low risk | Low risk | Low risk | Some concerns |
| Chen [28] 2023 | Low risk | Low risk | Low risk | Low risk | Low risk | Low risk |
| Dai [29] 2023 | Low risk | Low risk | Low risk | Low risk | Low risk | Low risk |
| Elahwal [30] 2023 | Low risk | Low risk | Low risk | Low risk | Low risk | Low risk |
| Krishnasamy [31] 2023 | Low risk | Some concerns | Low risk | Low risk | Low risk | Some concerns |
| Liu Z [32] 2023 | Low risk | Low risk | Low risk | Low risk | Low risk | Low risk |
| Orhon [33] 2023 | Low risk | Low risk | Low risk | Low risk | Low risk | Low risk |
| Toleska [34] 2023 | Low risk | Low risk | Low risk | High risk | Low risk | High risk |
| Yan [35] 2023 | Low risk | Low risk | Low risk | Low risk | Low risk | Low risk |
| Yu [36] 2023 | Low risk | Low risk | Low risk | Low risk | Low risk | Low risk |
| Choi [37] 2022 | Low risk | Low risk | Low risk | Low risk | Low risk | Low risk |
| An [38] 2022 | Low risk | Low risk | Low risk | Low risk | Low risk | Low risk |
| Ibrahim [39] 2022 | Low risk | Low risk | Low risk | Low risk | Low risk | Low risk |
| Menck [40] 2022 | Low risk | Some concerns | Low risk | Low risk | Low risk | Some concerns |
| Saravanaperumal [41] 2022 | Low risk | Some concerns | Low risk | Low risk | Low risk | Some concerns |
| Tochie [42] 2022 | Low risk | Low risk | Low risk | Low risk | Low risk | Low risk |
| Toleska [43] 2022 | Low risk | Low risk | Low risk | Low risk | Low risk | Low risk |
| Van [44] 2022 | Low risk | Low risk | Low risk | Low risk | Low risk | Low risk |
| Beloeil [45] 2021 | Low risk | Low risk | Low risk | Low risk | Low risk | Low risk |
| An [46] 2021 | Low risk | Low risk | Low risk | Low risk | Low risk | Low risk |
| Taskaldiran [47] 2021 | Low risk | Some concerns | Low risk | Low risk | Low risk | Some concerns |
| Shah [48] 2020 | Low risk | Low risk | Low risk | Low risk | Low risk | Low risk |
| Loung [49] 2020 | Low risk | Low risk | Low risk | Low risk | Low risk | Low risk |
| Hakim [50] 2019 | Low risk | Low risk | Low risk | Low risk | Low risk | Low risk |
| Toleska [51] 2019 | Low risk | Low risk | Low risk | Low risk | Low risk | Low risk |
| Shaman [52] 2019 | Low risk | Low risk | Low risk | Low risk | Low risk | Low risk |
| Gazi [53] 2018 | Low risk | Low risk | Low risk | High risk | Some concerns | High risk |
| Choi [54] 2017 | Some concerns | Some concerns | Low risk | Low risk | Low risk | Some concerns |
| Mogahed [55] 2017 | Low risk | Low risk | Low risk | Low risk | Low risk | Low risk |
| Subasi [56] 2017 | Low risk | Low risk | Low risk | Low risk | Low risk | Low risk |
| Hontoir [57] 2016 | Low risk | Low risk | Low risk | Low risk | Low risk | Low risk |
| Choi [58] 2016 | Low risk | Low risk | Low risk | High risk | Low risk | High risk |
| Bakan [59] 2015 | Low risk | Low risk | Low risk | Low risk | Low risk | Low risk |
| Hwang [60] 2015 | Some concerns | Some concerns | Low risk | Low risk | Low risk | Some concerns |
| Senol [61] 2015 | Low risk | Low risk | Low risk | Some concerns | Low risk | Some concerns |
| White [62] 2015 | Low risk | Low risk | Low risk | Low risk | Low risk | Low risk |
| Sahoo [63] 2015 | Low risk | Low risk | Low risk | High risk | Some concerns | High risk |
| Mansour [64] 2013 | Low risk | Low risk | Low risk | Low risk | Some concerns | Some concerns |
| Lee [65] 2013 | Low risk | Low risk | Low risk | Low risk | Low risk | Low risk |
| Techanivate [66] 2012 | Low risk | Low risk | Low risk | Low risk | Low risk | Low risk |
| Lee [67] 2012 | Low risk | Low risk | Low risk | Some concerns | Low risk | Some concerns |
| Lee [68] 2011 | Some concerns | High risk | Low risk | Some concerns | Low risk | High risk |
| Jung [69] 2011 | Low risk | Some concerns | Low risk | Low risk | Some concerns | Some concerns |
| De [70] 2010 | Some concerns | Some concerns | Low risk | Some concerns | Low risk | Some concerns |
| Ryu [71] 2009 | Low risk | Low risk | Low risk | Low risk | Low risk | Low risk |
| Salman [72] 2009 | Low risk | Low risk | Low risk | Low risk | Low risk | Low risk |
| Collard [73] 2007 | Low risk | Some concerns | Low risk | Low risk | High risk | High risk |
| Feld [74] 2006 | Low risk | Low risk | Low risk | Low risk | Low risk | Low risk |
| Shirakami [75] 2006 | Low risk | Low risk | Low risk | Low risk | Low risk | Low risk |
| James [76] 2005 | Low risk | Low risk | Low risk | Low risk | Low risk | Low risk |
| Hansen [77] 2005 | Low risk | Low risk | High risk | Low risk | Some concerns | High risk |
| Feld [78] 2003 | Low risk | Some concerns | Low risk | Some concerns | Some concerns | Some concerns |
| Curry [79] 1996 | Some concerns | Some concerns | Low risk | Low risk | Low risk | Some concerns |
| Katz [80] 1996 | Low risk | Low risk | Low risk | Low risk | Low risk | Low risk |
| Sukhani [81] 1996 | Low risk | Low risk | Low risk | Low risk | Low risk | Low risk |
| Tverskoy [82] 1994 | Some concerns | High risk | Low risk | Low risk | Some concerns | High risk |

Abbreviations: RoB, risk of bias. Assessment based on the Cochrane RoB 2.0 tool.
Low risk: Indicates low risk of bias; Some concerns: Indicates potential bias; High risk: Indicates high risk of bias.

Overall Bias reflects the highest level of concern across domains.
