# Supplementary figures and images for: Impact of the absence of opioid anesthesia on postoperative outcome indicators: a systematic review and meta-analysis
Source: Front Med (Lausanne). 2025 Aug 18;12:1639968. doi: 10.3389/fmed.2025.1639968 (PMC12399677; doi:10.3389/fmed.2025.1639968)

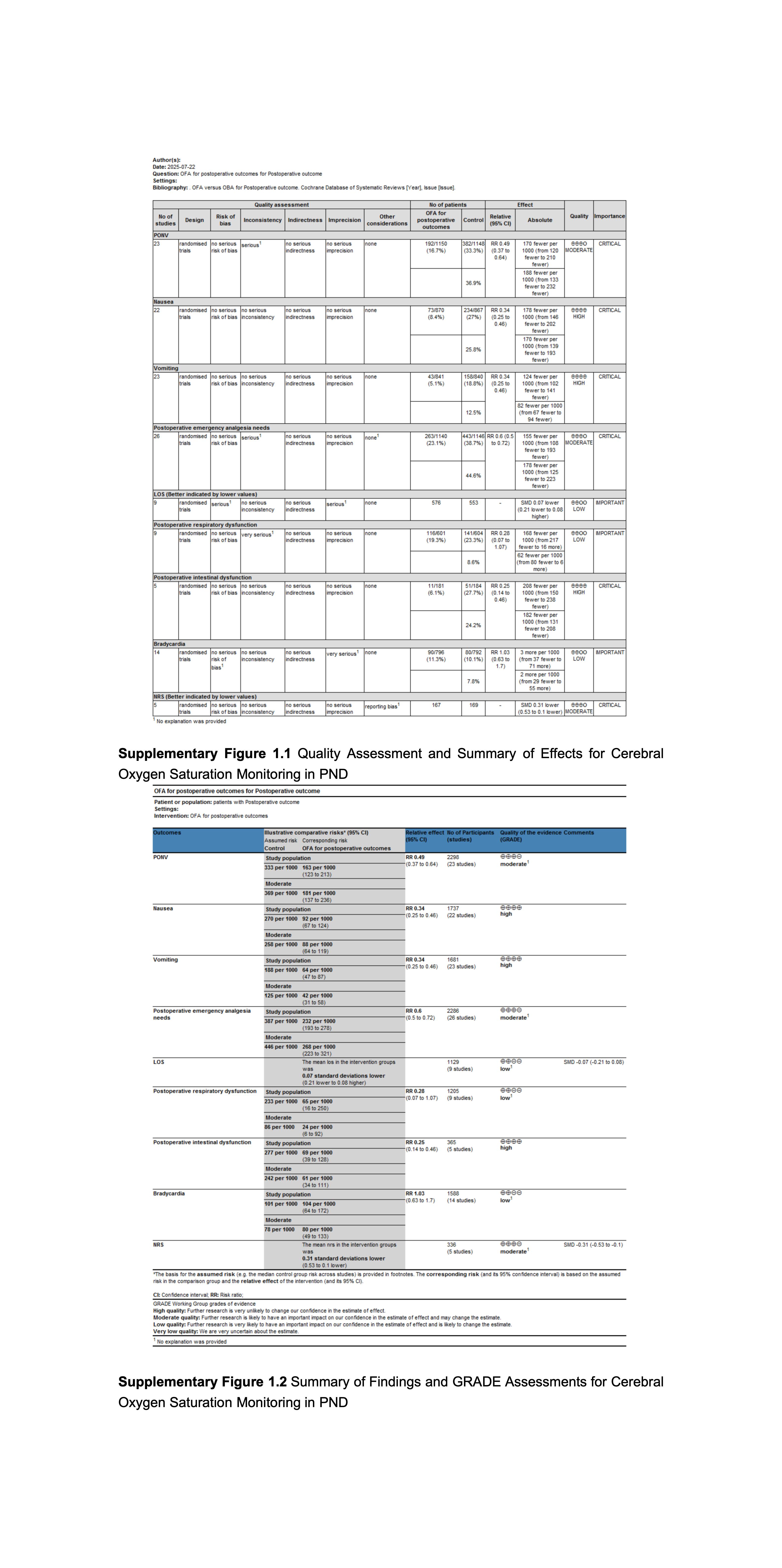

Supplement: Supplementary file 4 [file Image_1.JPEG]
